# Abstracts of papers presented at the 1995 Pittsburgh Conference

**DOI:** 10.1155/S1463924695000228

**Published:** 1995


Journal of Automatic Chemistry, Vol. 17, No. 4 (July–August 1995), pp. 123–160

### Abstracts of papers presented at the 1995 Pittsburgh Conference

The 1995 Pittsburgh Conference and Exposition on Analytical Chemistry and Applied Spectroscopy (PITTCON '95) was held from 5 to 10 March 1995 at the Ernst N. Morial Convention Center in New Orleans, Louisiana. The Pittsburgh Conference is the largest annual conference and exhibition on chemical instrumentation and laboratory equipment in the world. The conference ranks among the 50 largest tradeshows in the USA and attendance is in the region of 30 000 people.

PITTCON '95 featured an extensive exhibition of laboratory equipment and analytical instrumentation—over 1100 exhibiting companies occupied more than 3100 booths to display their latest product lines.

One of the outstanding features of any Pittsburgh Conference is the extensive technical programme. Over 1800 technical presentations were given in the areas of chemical analysis, spectroscopy, instrumentation and other topics. Portions of the programme were devoted to four institutes in the areas of bioanalytical chemistry, environmental chemistry, process analytical chemistry and quality. The institutes were designed to allow an attendee to concentrate on a specific topic within the larger PITTCON programme.

PITTCON '95 has been chosen by the United States Department of Commerce to be part of the Foreign Buyer Program (FBP). The FBP is designed to help American companies export their products and to aid international visitors at the conference.

The Pittsburgh Conference, sponsored by the Spectroscopy Society of Pittsburgh and the Society for Analytical Chemists of Pittsburgh, is a non-profit organization. The two Societies use the proceeds of the Conference to promote science education at all levels from elementary school to postgraduate training.

As in previous years, the editorial board of the journal is pleased to publish the abstracts of the presentations most relevant to the *Journal of Automatic Chemistry*. PITTCON '96 will be held from 3 to 8 March at McCormick Place, Chicago, Illinois. Details from The Pittsburgh Conference, 300 Penn Center Boulevard, Suite 332, Pittsburgh, PA 15235-5503, USA. Tel: (412) 825 3220 & (800) 825 3221; fax: (412) 825 3224.

### Automated sample preparation using the AutoChem workstation for LC and GC analysis

*Gerald L. Hawk and Paul F. Hensley, The Cardinal Instrument Company, Bristol, PA 19007*

The AutoChem Workstation brings together advances in the technologies of fluid control and computer software design for exceptional reliability and ease of use.

The system software, PrepWare, allows flexible and easily customized methods to meet whatever sample preparations requirements are necessary. Method development and system operation are ICON driven. The user needs no special training or automation experience to achieve accurate results. The AutoChem even has system self-validation and self-diagnostics to ensure reliable operation.

Precision and accuracy testing results for 'transfer' and 'addition' of liquids were presented. Typical performance for standard configuration systems allows reliable fluid movement plus or minus one microliter. LC and GC chromatograms for some typical applications were presented.

The AutoChem Workstation has many functional capabilities including: solid phase extraction, liquid/liquid extraction, membrane filtration, evaporation, mixing, internal standard addition, and instrument injection. Container flexibility is an important feature of the system, for example use of septum sealed vials or other specialized containers in existing applications.

### Determination of trace metals in process streams by capacitively-coupled microwave plasma atomic emission spectrometry

*Michael E. Rider, Scott R. Goode, Department of Chemistry and Biochemistry, University of South Carolina, Columbia, SC 29208; and Terry Layne, Beacon Technology, Inc., P.O. Box 608, Simpsonville, SC 29681*

The economical and immediate on-line analysis of process streams to determine trace metal concentrations is of great interest to industry. Conventional analytical methods for determining trace metals require samples to be removed from the stream, sent to a remote lab, and waiting hours to days for results. Capacitively-coupled microwave plasma (CMP) atomic emission spectrometry offers an inexpensive and efficient method for on-line and real-time monitoring of industrial processes, such as food, beverage, chemical, petrochemical, mineral production and environmental sampling. A CMP system capable of multi-wavelength spectral monitoring was described. Spectral features of the helium plasma and analytical figures of merit, such as detection limits, relative standard deviation

0142-0453/95 $10.00 © 1995 Taylor & Francis Ltd.

of analytical measurements, and accuracy of the method, were presented for various atomic species. The robustness of the plasma to matrix effects, such as high solids and foodstuff content, were demonstrated. Matrix effects in the CMP system were minimized by solvent evaporation and pre-ashing.

### The first intelligent electronic nose: a new analytical concept

*Laurent Moy and Jean Christophe Mifsud, Alpha M.O.S. SA, 3, Avenue Didier Daurat, 31400 Toulouse, France*

A new instrument was described (FOX 2000) which mimics the mammalian olfactory system using an array of six exchangeable semiconducting oxide sensors (there is a choice of 42 different detectors), combined with a specially developed pattern recognition software. The Fox 2000 can rapidly quantify and identify vapours. The sensitivity for certain kinds of chemical compounds can be in the low ppb level. A sample (solid, liquid, or gaseous), with the instrument's accurate flow control system, produces a finger print of the olfactory quality of a sample from the variation of each sensor. This is possible using unique statistical and Auto Neural Network (ANN) software. This artificial intelligence software permits the system to teach odours and patterns to the computer. After the learning phase, the Fox 2000 has the capabilities to recognize and quantify odours in seconds or minutes. It is complementary to GC and GC/MS.

After the system has been 'trained' for each application it can recognize the source of roasted coffee, the adulteration of cayenne pepper and other spices, the quality of beverages, the quality of chewing gum flavours, the putrefaction of fish or meat, the mould contamination of grains, the acceptance or rejection of mineral oil, and the quantity and quality of perfumes in cosmetic bases also seeing the differences in the base material in the cosmetic. It is also used in polymers for odours and paper packaging.

The system can be upgraded in terms of the number of sensors or kind of sensor technology depending on the level of descrimination needed. The expansion in number of sensors is accomplished in arrays of six sensors up to a total of 18 sensors.

The system can be used with a purge and trap sample system or with GC headspace auto samplers.

### Laboratory chemical inventory software

*Henry Nowicki, Barbara Nowicki, Homer Yute, Professional Analytical and Consulting Services, Inc. (PACS), 409 Meade Dr, Coraopolis, PA 15108; Paul Badger, Jack Pinkerton, Joe Hines, and Dave Clark, Geneva College, Computer Aided Engineering School, Beaver Falls, PA 15010*

It is good laboratory and business practice to know the kinds and amounts of chemicals and other resources in the workplace. The authors have designed and made available a simple streamlined but very useful relational database using FoxPro architecture that obtains this business practice. It is called *ProChem Inventory* software. This software runs on personal computers with 500 K conventional memory. The objective was to develop a software program capable of tracking the chemical inventory at a laboratory or small business using chemicals. This software is useful to comply with future governmental regulations, fire departments, insurance companies, purchasing departments and most important operating work groups can use the friendly software to improve their job performance. A simple and low cost and easy to use software program was developed to keep track of the chemicals in a laboratory. This new software uses chemical abstract service (CAS) numbers in addition to IUPAC common or generic names for chemicals. CAS numbers provide accurate retrieval of information from the data base.

The authors offered free copies to users who would work with this software now for evaluation. Feedback from users may improve its performance through upgrades.

### Computational methods for analysis of polymer infra-red data

*James L. Dwyer, Lab Connections, Inc., 5 Mount Royal Avenue, Marlborough, MA 01752*

Polymer systems demonstrate multivariate distribution of properties such as molecular weight, composition, and chain configuration. Commercial formulations are often subject to purposeful modifications of properties that impart desired end-use properties. Often these systems are polymer blends, and a variety of additives may be present.

The combination of chromatography and infra-red spectroscopy provides a versatile tool for characterization of polymers. The LC-Transform® an instrument which deposits the output of a chromatograph on an infra-red optical medium, is used for this purpose. The resulting data is a time-ordered set of spectra of the chromatogram.

The data set is large and complex, reflecting simultaneous variations in mass, composition of principal components, and configuration of chain elements. Spectral bands may be broad and overlapped. Computer analysis techniques are quite useful, even essential. Time is greatly reduced in extracting relevant information from the large data set. More insight is gained by examining a data set in various ways to extract differing information components.

Typical applications include:

(1) Analysis and identification of prepolymer constituents.
(2) Deformulation of complex systems such as adhesives, paints, and coatings.
(3) Compositional analysis of copolymers.
(4) Identification of polymer additives.

This paper presented a computer-based analysis of LC-IR data obtained on a variety of polymer samples. The LC-IR data sets are first visually arrayed as a three-dimensional IR map of a chromatogram. Various functional group 'chemigrams' are extracted and mathematically combined to provide useful compositional distribution functions. The data is also presented in a 2D correlation format to define those spectral features that vary synchronously and those that vary independently, highlighting features not readily observable with one-dimensional spectra. Statistical regression techniques, which can extract quantitative information from overlapping bands and noisy data sets, were also illustrated.

### Automated method validation, system suitability testing, and quality control in the routine

*Ludwig Huber and Mike Thomas, Hewlett-Packard GmbH, Hewlett-Packard Str., 76337 Waldbronn, Germany*

Regulations such as Good Laboratory Practice (GLP) and Current Good Manufacturing Practice (cGMP), quality standards and guidelines, such as EN45001 and ISO Guide 25, require that analytical methods be validated prior to routine use. Systems should be tested for suitability before and during routine use and quality control checks should be built into routine analysis to verify analytical results for accuracy.

This paper presented criteria for method validation and system suitability testing in gas and liquid chromatography and in capillary electrophoresis. The authors gave guidelines on how to automate the entire process for highest efficiency and compliance with the various regulations and quality standards. The most critical parameters, such as method detection and quantitation limits, selectivity, accuracy and ruggedness were discussed in detail.

Guidelines were also given on how to select, verify and use (certified) reference material for quality control checks.

### Semesters in cyberspace: the virtual college at NYU

*Richard Vigilante, New York University, 48 Cooper Square, New York, NY 10003*

Corporate training is a $100 billion a year business in the United States. Upwards of 35 million individuals receive formal, employer-sponsored education each year. The facilities, staff and travel costs of traditional training programmes are considerable. To meet the continuing demand for new skills, education has to be constantly available to employees through convenient and economical means. The cost-effectiveness of online versus on-site training is of increasing interest to thousands of business and public organizations.

New York University's Virtual College teleprogram is an online system for the efficient production and delivery of a wide range of college, business and technical courses over data networks to home or office PCs. Through the Virtual College, students receive instruction, ask questions, conduct analyses, resolved problems, and complete projects—all largely at their own convenience and from practically anywhere in the world. Telecourse delivery is via Lotus Notes, a powerful group information manager that gives people who work together an electronic environment within which to collect, organize and share information, using networked PCs.

This paper described the teleprogram's use of 128 kbps Integrated Services Digital Network (ISDN) lines to deliver digital videocourses to student PCs equipped with sound cards and ISDN adapters. The teleprogram utilizes VideoNotes, a new video storage and delivery system that allows users to capture and embed video clips in Notes applications and databases. With VideoNotes, students can activate icons located in various telecourse lesson, case study or text documents and have instructional video clips sent from the teleprogram server to their PCs. The Virtual College is a unique distance education programme where all instructional materials—video, lectures, laboratory projects, and readings—are digital and interactively accessible through one common user interface.

### Strategic knowledge management and the virtual workgroup

*Gregory Möller, Analytical Sciences Laboratory, University of Idaho, Moscow, ID 83844-2203*

Organizational knowledge, an asset with a high primary value, is often poorly managed. This is particularly true in larger organizations. This knowledge can be operational, historical, interpretive and the knowledge products of inquiry, experimentation, discovery and learning. The absence of a global and active knowledge management plan in any organization may be a threat to continued existence in the competitively required 'do more with less' environment. In leaner and flatter organizations, productivity, employee empowerment and organizational success can result from a knowledge management strategy that allows for enhanced access to the information arterial of the organization.

Strategic Knowledge Management (SKM) is the use of the virtual platform allowed by telecommunicating computers to support heterarchical processes, knowledge economy, knowledge archival and daily communication in a distributed workgroup.

Invoking this virtual platform is essential to any contemporary SKM plan. The formation of virtual workgroups is now possible through innovative advances in groupware—software designed for group interaction and wide area networks, as well as the interaction afforded by Internet. This paper described some future considerations and some current applications of SKM in organizational support.

### A fibre optic coupled Raman microscope for near-line process control

*Kevin L. Davis and Joseph B. Slater, Kaiser Optical Systems, Inc., 371 Parkland Plaza, Ann Arbor, MI 48106*

The authors described the development of a Raman microscope system designed for near-line process control. The system circumvents many of the alignment difficulties encountered with traditional Raman microscopes. This is accomplished by coupling a compact integrated process Raman system to an optical microscope through the use of fibre optics. The Raman microscope provides chemical information with a high degree of spatial resolution. The microsampling nature of this technique makes it ideal for identifying localized process imperfections.

While the Raman microscope finds research application in a wide range of fields including biology, polymer chemistry, catalysis, geochemistry, and semiconductor analysis, the use of this technique for process control applications has been hindered by equipment complexity and instability. The implications of fibre coupling on system stability and performance were discussed. Experiments evaluating system throughput and spatial resolution will be detailed. Polymer laminates, semiconductor wafers, and carbon fibres were among the samples presented. Recent progress towards process applications were also discussed.

### The use of FTIR-microscopy in the detection of chemicals on skin

*Herbert J. Kaiser and Walter K. Gavlick, Calgon Vestal Laboratories, P.O. Box 147, St. Louis, MI 63166*

Many types of chemical agents are applied to skin to prevent or treat infections. The fate of these chemicals on the surface of the skin has long been a question posed by formulators and regulatory agencies alike. The ability to detect these agents on the skin, both to initially verify their presence and also to determine the length of time that they are there, has been very difficult. Pig skin has been used as a model of human skin for studying the activity of antimicrobial agents. The combination of FTIR-microscopy and pig skin models has led to the development of techniques suitable for the detection of these chemical agents on the skin surface. This technique was described along with examples of the detection of various antimicrobial agents.

### Controlled potential generation of arsenic hydrides with detection via ICP/MS

*T. H. Ridgway, K. Khalema, L. Olsen and J. A. Caruso, Department of Chemistry, University of Cincinnati, P.O. Box 210172, Cincinnati, OH 45221*

The controlled potential electrolysis of arsenous acid and methyl arsenic acid at platinum, gold palladium, and nickel electrodes in a flow cell has been investigated as a means of hydride generation. The flow cell is constructed from stainless steel. The electrodes are thin metal foils bonded to the steel substrate with Terfzel. The Terfzel is placed between the two metals under mechanical pressure and then the sandwich is heated in a vacuum at 350°C for 20 minutes. The resulting bond is tough, chemically inert and electrically insulating. The flow chambers are formed from thin plastic spacers between a Nafion ion exchange membrane. This arrangement forms isolated working and auxiliary electrode compartments.

The gaseous products from electrolysis in the working compartment are introduced into an inductively coupled plasma-mass spectrometer (ICP/MS) through a highly porous teflon tubing based gas-liquid separator. The results are compared to those obtained with cyclic voltammetric studies at the same type of electrodes. A metallic arsenic electrode has also been employed for cyclic voltammetric mechanistic studies.

The electrogeneration—ICP/MS method provides a sub ppb detection limit. The best overall performance was obtained with the gold and palladium electrodes. Nickel and platinum appear to produce a higher hydrogen to hydride ratio which leads to instability of the ICP plasma.

### Chromatography with ICP-MS, the next steps

*Robert C. Hutton, Darren Mennie and Paul Sigsworth, Fisons Instruments Elemental Analysis, Ion Path, Road Three, Winsford, Cheshire CW7 3BX, UK*

The facility to perform on-line chromatographic measurements with ICP-MS is becoming an ever more important development area. It provides three important benefits to the user: the capability to perform matrix removal on-line; the capability to perform speciated measurements; and additionally the use of on-line column technology can be used to pre-concentrate elements to allow even lower detection limits to be achieved. Each of these areas was explored in this paper.

On-line pre-concentration lends itself to the analysis of actinide elements where not only is there a need to handle small amounts but also there is a necessity to quantitate to below ppq levels. In the work discussed in this paper, combining an enhanced sensitivity ICP-MS instrument with a micro-column pre-treatment stage was seen to yield detection limits of below 0.1 ppq for isotopes of $Pu^{239}$, $Np^{237}$ and $U^{235}$.

These enhanced sensitivity instruments can also be used effectively to quantitate ultratrace elements in heavy matrices such as sea water. Once the matrix has been removed on-line the full capabilities of these instruments can be used and data were presented outlining these procedures.

Lastly, performing speciated measurements on-line opens up new possibilities when combined with the isotope capabilities inherent with ICP-MS and this was discussed in detail.

### Recirculating loop FI manifold for sample and standard dilution for ICP-MS

*Honghong Ge, Julian F. Tyson, Chemistry Department, University of Massachusetts, Box 34510, Amherst, MA 01003-4510 and Eric Denoyer, Perkin-Elmer Corporation, 761 Main Ave., Norwalk, CT 06859*

A recirculating loop manifold has been developed for on-line dilution sample introduction for ICP-MS, to construct a calibration curve from a single standard and to (re)analyse samples by successive dilution to reduce the matrix effect (a requirement for many EPA ICP-MS methods). The theoretical dilution factor of the manifold is the ratio of the total loop volume to that of the loop which does not form part of the injection valve. The loop volume was determined by a photometric method with tartrazine as the solute. External and internal calibration curves with 1.000 and 0.997 correlation coefficient have been constructed. The dilution behaviour of elements which can be determined by EPA 200.8 was examined. Most of them exhibit linear dilution behaviour with good precision from about 100 ppb to the EPA 200.8 total recoverable detection limits, a procedure to recover all forms of analytes in a sample. The dilution factors of some elements such as Hg, Bi, Mo kept decreasing with the successive dilutions. This may be due to the adsorption of these analytes on the tubing wall. The manifold was optimized by following the concentration oscillation with a UV-VIS detector inserted inside the loop. Mixing devices such as mixing chamber and split-confluence were studied to speed up the mixing of the remaining sample and the diluent. Spike recovery versus on-line dilution was studied for complicated matrices such as wine.

### Post-column electrochemical reduction reactor for fluorescence detection of aldehydes using HPLC

*M. J. Patrick and J. H. Mike, Department of Chemistry, Youngstown State University, Youngstown, OH 44555*

A commonly encountered difficulty in HPLC analyses is the inadequate detectability of analytes in the eluent and overlapping bands resulting in poor peak resolution. These problems make separation, identification and quantification of aldehydes difficult. UV absorption, one of the most common detection modes, is most problematic in this regard. In an attempt to explore remedies to this problem for aldehyde analysis, a post-column electrochemical reactor system was developed with the hope of coupling the device to post-column fluorescence detection of these compounds. The fluorescence derivatization ultimately proved unacceptable for aldehyde determinations, however. Fortunately the electrochemical reactor itself, combined with UV detection of aldehyde-2,4-dinitrophenylhydrazine (2,4-DNPH) conjugates, proved to give good specificity for determination of aldehydes. Using an electrochemical reactor for reduction ensured completion and reproducibility of reaction and eliminated the need for post-column reagents or large post-column reactor columns. Also, concerns of eluent pH were eliminated, since the reaction occurred after the pH-sensitive column. In this study, a mixture of aldehydes was derivatized with an acidic solution of 2,4-dinitrophenylhydrazine prior to injection onto the chromatographic column. The mixture was separated using a reverse-phase column and a 55% acetonitrile-45% water mobile phase containing 0.01 M sodium perchlorate.

The on-line electrochemical reactor was used for reduction of aldehyde-2,4-DNPH conjugates to aldehyde-2,4-diaminophenylhydrazine conjugates. The reactor was a two electrode system with zinc working and stainless steel auxiliary electrodes. The reduction was monitored as the shift of the wavelength maxima from 345 nm (2,4-DNPH conjugates) to 212 nm (2,4-DAPH conjugates).

Simultaneous chromatographic plots, obtained by monitoring absorbance at 212 nm and 345 nm using a diode array detector showed similar behaviour. The advantages of this method are improved specificity of analysis with little effect on detection limits.

### An on-column concentrator/reactor for the trace analysis of peptides in capillary electrophoresis

*Louann Cruz, Kerri Seggebruch and Jonathan V. Sweedler, Department of Chemistry, University of Illinois, Urbana, IL 61801*

The ability to detect trace levels of peptides in physiological samples is a daunting task in biochemical research. The authors' laboratory is investigating the technique of high performance capillary electrophoresis (HPCE) to accomplish this task.

Currently, the lowest limits of detection for peptides in capillary electrophoresis are achievable using laser-induced fluorescence. However, due to the slow kinetics and many competing side reactions, trace levels of peptides do not derivatize well with fluorophores. Therefore, specialized capillaries are used to detect trace level peptides in HPCE via on-line preconcentration and derivatization.

These preconcentrator capillaries consist of a small plug of reverse phase packing material at the injection end of the capillary. The packing material is first saturated with a derivatizing agent such as NDA under conditions that maximize the hydrophobic interactions between this reagent and the support matrix. Excess derivatizing agent is then washed away. Next, trace level peptides, contained in a high salt buffer such as is found in physiological samples, are injected onto the capillary where derivatization rapidly occurs. Once derivatized, the peptides are eluted from the column section of the capillary by using a low salt buffer containing an organic modifier. As the derivatized peptides elute, they enter the main section of the capillary where they are electrophoretically separated.

The advantage to this approach is that both derivatization and detection of peptides is on-line. The separation of a series of peptides concentrated and derivatized using this method was presented.

### Derivatization and high sensitivity HPLC analysis of amines using a fluorescent reagent, 6-aminoquinolyl-N-hydroxysuccinimidyl carbamate

*Steven A. Cohen, Igor Mechnikov and Charlie van Wandelen, Waters Corporation, 34 Maple St., Milford MA 01757*

Derivatization methods in analytical chemistry are an important tool for the analysis of reactive species, particularly amines, which may lack easily detected functionalities. The authors recently developed a new reagent, 6-aminoquinolyl-N-hydroxysuccinimidyl carbamate (AQC, Waters AccQ·Fluor$^{TM}$ reagent), that reacts with both primary and secondary amines and brings simplicity to the derivatization procedure while providing superb linearity, reproducibility and accuracy for amino acid analysis. This paper described the analysis of a number of other target amines that have now also proven amenable to derivatization by AQC, retaining many of the same advantages previously demonstrated for amino acids. For example, the analysis of amine drugs, can be significantly enhanced by the labeling procedure. The selectivity and sensitivity afforded by the fluorescent tag is often highly favourable for analytes in a biological matrix. Polyamines, such as putrescine and spermidine, are also amenable to derivatization-enhanced detection. Derivatization of peptides allows for ultra sensitive detection below 1 nM concentration. Most samples require little or no sample cleanup, and derivatization can be carried out simply by buffering the sample and adding reagent. Reagent removal is not required and the derivatized samples are usually injected with no further preparation. Typical detection limits range from 50–500 fmol. Relative standard deviations are 1–4% for standards and 2–7% for samples.

Methods for the quantification of the secondary amine drugs ephedrine and pseudoephedrine in plasma were shown as examples illustrating the ease with which real samples can be analysed. Despite low parts per billion concentrations, sample work up remains straightforward, and can be done simply via protein precipitation and an optional concentration step.

### Certification and accreditation of reference materials suppliers

*Alan Squirrell, National Association of Testing Authorities, Australia, 7 Leeds Street, Rhodes, NSW 2138, Australia*

At a time when the international community is becoming increasingly aware of the importance of traceable and comparable chemical measurements, the focus is on increasing the number of high quality (certified) reference materials that are available to laboratories. If chemists are serious about putting chemical metrology on the map (and recent developments through international groups like ISO/REMCO, CIPM and CITAC suggest they are), it is not only essential that we can form some consensus agreement on the fundamental concepts involved (i.e. traceability, uncertainty and fitness for purpose) but also grasp the important role that reference materials must play. Without these, it will be impossible to establish secure traceability chains.

The demand for high quality reference materials inevitably leads to the need for a scheme for the certification and accreditation of reference material suppliers and certifiers (rather than any form of 'self-declaration'). Moves towards this have already occurred, including the suggestion by ISO that an International Accreditation Board be established.

However, the conformance assessment infrastructure already exists in many countries to provide national schemes and a few certification and accreditation bodies are already operating independent programs based on third-party (peer) group assessment of reference material producers. To be effective such schemes must contain two elements—certification of the producer's quality system (for example, based on ISO 9002) and also accreditation, a formal recognition of technical competence. This accreditation could be based on existing ISO Guides 25 and 30–35 together with supplementary criteria for specific types of reference materials.

The author argued that a transparent scheme, based on trust and co-operation between relevant national and international bodies (leading to mutual recognition) is now essential.

### Electronic laboratory notebooks, workgroup systems, and object-oriented LIMS in R&D

*Rich Lysakowski, Optimize Technologies, 8 Pheasant Avenue, Sudbury, MA 01776*

Laboratory Information Management Systems (LIMS) have fallen short of the vision promoted shortly after their introduction in the 1980s. As implemented by most vendors, they are simply relational database systems that can use only simple datatypes. They do not handle most of the more complex laboratory datatypes, such as microscope images, 3-D tomography films, chemical structures, spectra, documents, spreadsheets, and others. Current commercial LIMS also do not handle many complex tasks that occur in the laboratory, such as data collection, integration and access of data from all data sources, project and equipment scheduling, direct access to on-line information services, or workflow processes such as structured routing, witnessing, approval, and distribution of data and reports. A trend of most LIMS vendors is to redesign these products to be 'object-oriented', because this helps the database deal with more complex data such as spectra and chemical structures, and build more sophisticated applications for laboratory processes.

While this relatively new 'object-oriented' paradigm for software can give significant benefits to software developers, object-oriented software requires large investments in design and usability testing to give real benefits to users. Recently introduced software, such as Lotus' Notes, Digital's LinkWorks, the ForeFront Group's Virtual Notebook System, and Helix Systems/Megalon's Research Station, and Documentum's EDMS application systems, are all finding application in R&D. These softwares fit in different niches and all have their 'sweet spots'. Knowledge of these sweet spots is critical to finding appropriate applications for them. Gaining this knowledge can take years, which is not possible in most laboratory situations. The author presented results and personal experiences from several case studies in research, development, and application of them, and then presented a comparative analysis to summarize their optimal application domains.

### Extending the laboratory into a TQM process environment

*Michael A. Tyszkiewicz, Industry Sector Manager, Process Industries, Automated Compliance Systems, Inc., 245 Hwy 22 West, Bridgewater, NJ 08807*

The process industry is undergoing radical changes because of customer-driven requirements for lower prices and higher quality. The 1980s brought about quality initiative, continuous improvement, and ISO-9000 programs that focused on industry practices and methods. Process automation systems focused strictly on process variables and process control, while Laboratory Information Management Systems (LIMS) traditionally focused on samples and data strictly from the laboratory perspective. Functionality was developed in both of these systems to meet end-user workflow requirements and increase efficiency. The linking of lab data to the process area—functionality required to make instantaneous process decisions—has not been adequately addressed.

While relational database products, such as Oracle, have made the exchange of information much easier, current available LIMS technology falls short of total process integration. LIMS packages use status changes of samples as triggers to send messages and data to other systems. In order to track data, samples and tests are assigned a status once they arrive in the laboratory. Some of the common status descriptors are pre-logged, logged (received in the laboratory), completed and authorized. LIMS vendors then write routines that trigger site-specific functional reports or actions at status changes. These actions may be as simple as sending data in file form for another system to read at sample completion, or as complex as running product-grading routines. This strategy assumes all product quality data is owned and maintained by the lab. However, this assumption only holds true at a small number of installations.

The total integration of a process lab with the processing area involves more than just the exchange of in-lab data in a timely fashion. In an area where several process steps are involved in production, there are many interactions between the lab and process area, and many human tasks and decisions affect product quality and operational efficiency. Most of these take place without computer assistance or electronic documentation. Simple visual inspections of product, or physical and visual testing performed in the process area, are critical data points that result in approval to continue the next process step. The lab and process area must be integrated to work as one unit with an ultimate goal of high product quality and maximum efficiency. The goal will only be achieved, however, when process area data and procedures that affect product quality are integrated with in-lab data to allow statistical analysis of these variables for process optimization.

### Networking to improve quality control for VOA data

*Michael J. Herdlick, Aqua Tech Environmental Labs, Inc., 6878 South State Route 100 P.O. Box 76, Melmore, OH 44845*

Over the years, Aqua Tech Environmental Laboratories, Inc., has specialized in the analysis of Volatile Organic Analytes (VOAs) in drinking and waste waters by US EPA Methodology. With the tremendous advances made in both the GC/MS equipment and in network computerization, it has been difficult to collect and to process data in a centralized environment such that quality control reports and VOA data packages can be generated for clients. ATEL, however, has taken steps to produce results and to examine quality control data in a timely and efficient matter.

Through the use of a commercial LIMS, it has been possible to network the VOA GC/MS systems. The network has given the flexibility to process data and to generate US EPA CLP forms for clients, all this from one centralized network server area. Finally, it has been possible to develop a systematic program to archive the raw data without having each chemist archiving from their respective LAN area.

At ATEL, this system is currently being developed and utilized on a micro scale with the hope of expanding eventually to cover the entire lab. This presentation demonstrated how a commercial lab is networking to not only improve quality control data, but to create data packages for clients and to archive data.

### LIMS in the manufacturing environment

*Mary D. Hinton, Applied Computer Systems, 3540 Country Ct. N., Mobile, AL 36619*

The type of laboratory that is considering a LIMS implementation often determines the goals of a LIMS. A manufacturing laboratory has a very different approach to managing lab information than a testing services laboratory. For example, in a testing service laboratory the cost of each test performed is a major consideration in company profitability, whereas the cost of a test in a manufacturing environment is usually miniscule compared to the price of producing the product itself. So schedules, worklists, and other cost reducing measures are very important in a testing service lab, but are not the primary functions for a manufacturing LIMS.

The manufacturing laboratory's function is to contribute to the overall success of producing and selling quality grade products. Today's manufacturing laboratory provides information to aid in process control decisions and to determine which products can be sold to which customer.

The two primary objectives of a manufacturing quality assurancy laboratory are to provide: assurance of product specifications; and statistical process control information.

The manufacturing lab provides product information to other departments within the company during the various stages of the product life cycle. Some departments that depend on lab results are:

(1) Shipping/receiving.
(2) Material handling/inventory.
(3) Ordering/scheduling/planning.
(4) Process control.
(5) Sales/customer service.
(6) Environment/waste management.
(7) Management information systems (MIS).
(8) Quality assurance.

Each of these departments was discussed in this presentation with respect to current interaction with the laboratory and what departmental needs should be met in a manufacturing LIMS.

Besides the generic functions provided by most LIMS, such as automatic data calculations, security, reports, and graphs, a manufacturing LIMS should provide special functions including revision control for test methods and specifications, the ability to grade a product, generation of COAs (certificates of analyses), and other manufacturing requirements.

Any LIMS contributes to the quality assurance of laboratory data results, but a manufacturing LIMS can also improve the quality of the products themselves. During the various stages of a process, samples are tested by the laboratory. The LIMS can collect, interpret, and distribute the results to other departments, such as process control, shipping, and inventory.

### Automated evaluation of infra-red spectral quality in quality control applications

*Joe M. Hodkiewicz and Dean E. Roberts, ATI Instruments, 1001 Fourier Dr, Madison, WI 53717*

Dedicated analysis methods used in quality control laboratories are often executed as macro programs in an effort to reduce variation in the method. While many aspects of the analysis can be automated to deliver specific answers for a particular sample, sample preparation and introduction remain highly operator dependent. Sample preparation and introduction cannot be fully controlled in a macro, but the resultant infra-red spectra have information which can be used to evaluate the overall quality of the spectrum, sample preparation, and sampling accessories used. This paper presented methods amenable to automation for determining the quality of sample preparation and sampling accessory performance for transmission, ATR and diffuse reflectance analysis.

### On-line spectroscopic analysis of dairy products for quality control

*John Richmond and George Asimopoulos, LT Industries Inc., 6110 Executive Blvd #800, Rockville, MD 20852*

Dairy products are typically formulated to meet a specific composition for consistent quality. Properties like pH, solids content, percentage fat and percentage protein need to be closely controlled. Near infra-red (NIR) spectroscopy has been used to quantify the composition of liquid dairy products on-line in an industrial environment and to monitor and control faster and with consistent quality (QC & QA).

Advantages of the NIR analysis are that it is fast, nondestructive, automatic, and clean. Fibre optic technology combined with optical multiplexing allows several process streams to be monitored with a single spectrometer, reducing the cost per channel. Calibration models based upon partial least squares algorithms are robust and accurate.

Faster quality control and continuous monitoring of dairy products increases the end-product's quality and NIR analysis provides a fast, cost-efficient and reliable measurement technique. This paper presented case studies, customized configurations for the dairy industry and described quality improvements achieved through NIR techniques.

### On-line fluorescence determinations of near-IR dyes separated by capillary electrophoresis

*Benjamin L. Legendre Jr. and Steven A. Soper, Department of Chemistry, 232 Choppin Hall, Louisiana State University, Baton Rouge, LA 70803*

DNA sequencing typically requires a set of four unique probes to identify the terminal base during the separation process. When fluorescence is used as a detection scheme, spectral differences in the emission wavelengths of the probes allow separation to be performed in a single lane but require multi-wavelength excitation and multiple detection channels. To circumvent this problem, a single lane, single fluor sequencing strategy has been developed which involves lifetime discrimination of a set of four unique near-IR dyes.

Investigations have begun toward evaluating the feasibility of determining the fluorescence lifetimes of near-IR dyes separated by capillary electrophoresis (CE) at trace levels using time-correlated single photon counting. Exponential decay profiles were constructed from the emission of the dye molecules and lifetimes determined using simple algorithms. The lifetimes obtained from the CE studies were compared to the individual lifetimes using static measurements. This discussion focused on presenting results on determining lifetimes for a series of near-IR fluorescent dyes during capillary electrophoretic separation. The precision and accuracy of the determinations was described as well as the instrumentation necessary to make such determinations.

### Remote diagnosis as an effective and inexpensive means of instrument support

*D. W. Messaros, K. D. Rohn and G. M. Wilson, Hewlett-Packard Company, 2850 Centerville Road, Wilmington, DE 19808*

Analytical instruments typically provide minimal access to control parameters that are useful for instrument diagnosis. By developing software to access these parameters, one can remotely diagnose and often fix instruments in a timely and cost effective manner. This paper described how remote control of key instrument parameters by a personal computer can facilitate this approach to support.

Method verification and optimization are possible from a remote personal computer. Once a chromatographic expert remotely connects to an instrument, method verification is a straightforward task. The chromatographic expert can optimize the method per a local chemist's requirements.

The authors demonstrated remote diagnosis and repair of instrumentation. This capability further allows automatic logging of maintenance procedures and repairs required by GLP or ISO. They presented the architecture of their remote support tool and discussed its key elements.

### The automated analysis of soil samples using a relational database management system (RDMS)

*Walter R. Anderson, Weston Environmental Metrics Inc., University Park, IL 60466*

Percent solids analysis is required for the determination of all organic and inorganic compound concentrations in soil samples. The gravimetric method includes manually recording weights, calculating results, and re-typing data to transfer to a laboratory information management system. These steps are tedious and error prone.

The percent solids method was modeled with a relational database management system. A RDMS is an effective tool to rapidly model analytical methods. This software development platform provides efficient storage and effective modelling of data along with tools for screen design and data reporting.

The automated system is used to capture weights from a balance, calculate percent solids results, import sample data from the LIMS and export sample data and results to the LIMS. As a result, the logbooks for this method have been eliminated and both the time of analysis and reported errors have been significantly reduced.

This paper described the process of modelling the analytical method and the software design principles used. Performance data used to validate the automated system and to document the time savings were also presented.

### Application of computer modelling to thermometric titration data

*John A. Lynch and Russell P. Linnemann, II, and Eric T. Lane, University of Tennessee at Chattanooga, Chattanooga, TN 37403*

Thermometric titrations allow examination of reactions through their resultant temperature changes. Unfortunately, the nature of heat assures obstruction of this view by thermal noise from a variety of sources. To overcome this limitation the authors developed a completely automated system. A Macintosh computer controls this experiment, acquires its data and supports data analysis.

The overall analysis protocol was the main focus of this paper. Its first step is to use input from the experimental control loops and the operator's inspection of the titration curve to assign boundary locations between reaction and non-reaction regions. Second, extraneous background effects are removed from the reaction region. Third, the reaction region is reduced to a scale of zero, for no reaction, to one, for complete reaction. Fourth, these data are fitted to chemical algorithms using nonlinear regression analysis; the software 'proFit', produced by Quantum Soft, was chosen for this task because it uses efficient Marquardt-based routines and allows for incorporation of iterative approaches in the algorithms selected for the fit. Finally, the method of deviation pattern recognition, pioneered by Louis Meites, is used to judge the quality of the match of the experimental data to the algorithm; thus allowing a cycle of continuous refinement.

This approach reduces the operator's job to: selection of the chemical system for study; providing answers to prompts; choosing appropriate algorithms and adjusting them as needed to produce the best match to the data. Examples were given of how relative novices are able to examine a variety of chemical systems and find significant information, such as values for $K_{eq}$, $\Delta H$, $\Delta G$, $\Delta S$ and $k_f$ and $k_b$.

### A LAN-based solution for data acquisition in a multi-vendor laboratory

*David B. Tucker and Kathleen A. McGrath, Hewlett-Packard, Analytical Products Group, Palo Alto, CA 94304*

A growing trend among analytical instrumentation has been the use of data acquisition systems connected via standard Ethernet LANs. Such systems offer centralized control for dozens of instruments while eliminating the need for dedicated, proprietary network connections within the laboratory. A common limitation, however, has been the inability to directly control heterogeneous instruments originating from multiple vendors.

A solution to the problem is offered by the HP 35900E Dual Channel Interface in combination with the HP ChemServer 4930 software. The system uses a customizable device configuration methodology to allow support of a wide range of instruments. The HP ChemServer software provides the interface through which device control parameters are specified and integrates that interface with its sequencing module. Only an ASCII configuration file and the actual device control module (a UNIX shared library, similar to a Microsoft Windows DLL) are different for each device.

The device configuration files standardize the relevant attributes of a particular type of analytical instrument: GCs, LCs, or autosamplers. This standardization allows for a uniform user interface across products from different vendors. At the same time, the configuration file allows users or developers to support the unique features of each product with different labels and valid ranges for the instrument parameters. GC autosamplers, for example, are abstracted as simply a fixed number of injectors with an independent set of parameters for each injector. The names and functions of the parameters may differ between autosamplers from different vendors, but such differences can be easily described in the configuration file.

The HP ChemServer sequencing software standardizes the actual control of the device by providing a fixed applications programming interface (API) for each type of instrument. The entry points are high level operations, such as DownloadDevMethod to send a temperature program to the GC or DoAlsInjection to trigger the autosampler. The modules with these functions are supplied by HP or third-party developers and are dynamically accessed as the sequence is running. Each command is implemented using standard C language functionality. Additionally, an interface library is provided to simplify access to the configuration file and the file that contains the user specified parameters. Functions to send and receive commands via the HP 35900E's serial communications ports are also provided.

The presentation focused on the details of the configuration files and the device control modules. A complete description of the steps required to integrate a new instrument was given.

### Windows WOSA: a new standard for test instrument integration

*Patrick E. Dessert and Christian C. Wagner, SA Consulting Company, 39526 Westminster, Novi, MI 48375; Carol J. Kelly and Donald E. Lee, Ford Motor Company, Ford Central Laboratory, 15000 Century Dr, Dearborn, MI 48120*

The predominant trend in test instrumentation control and interface platforms is the use of Microsoft Windows on a 486+ class PC. Currently there is a great deal of effort in the integration of laboratory equipment to LIMS systems. The most efficient answer to this problem is the use of a Microsoft standard toolkit which resides on top of Windows. This is called the Windows Open System Architecture or WOSA. WOSA is a computing standard developed by the Microsoft Corporation for the integration of 'networked' computing elements. Major components of WOSA include:

(1) Mail Application Programming Interface (MAPI)—The standard for endowing applications with the ability to communicate using e-mail.
(2) Windows Sockets (WINSOCK)—The standard for endowing applications with a common set of socket calls to communicate through network sockets.
(3) Open Data Base Connectivity Standard (ODBC)—The standard for endowing applications with a common way of connecting to a database server.

Today, there is a preponderance of unneeded effort in devising standards for laboratory instrumentation to share data. With the WOSA standards these efforts are totally dilatory. Instead of worrying about trying to decipher the data file of an arbitrary machine for integration to other lab and business efforts, WOSA provides the capability to 'encapsulate' the lab instrument from the rest of the laboratory.

The key is that through the use of WOSA, other laboratory systems could communicate with the instrument in a client/server type manner and ask for the results of a test to be returned in a particular format. For example a LIMS system can ask the instrument to return the test results as an EXCEL spreadsheet.

Communications to the instrument do not need to be limited to the acquisition of data alone. Several classes of messages to be sent to the instrument have been devised including: Configuration, Archiving/Backup, Quality Control, Preventive Maintenance, and Data Retrieval.

In the paper the authors presented a computer topology and set of messages to guide instrument integration. It is believed that the adoption of these tools will move the analytical instrumentation industry away from developing functionality that is already available and move them to decreasing instrument cost and increasing instrument functionality.

### Moving lab data to a life-cycle and standards-based approach

*Rich Lysakowski, Optimize Technologies, 8 Pheasant Avenue, Sudbury, MA 01776*

Efficient laboratory data collection is possible without data standards. However, that is where problems begin when standards do not exist. And the problems never end through all subsequent uses of the data. The objective of the ADISS Standards Program is to create a life-cycle approach based on standards to ameliorate problems for data capture, communication, storage, and archival. The Analytical Instrument Association (AIA) is using the analytical data model and software tools recommended by the ADISS Program in 1990. The AIA member companies, which include all the world's major instrument companies and many smaller ones, have now implemented these data standards for chromatography and mass spectrometry. The AIA is now finishing a standard for infra-red and UV-Visible spectroscopy. The Laboratory Automation Standards Foundation (LASF), is developing a data standard for all major branches of flame and plasma spectroscopy; it will be based on the ADISS Analytical Information Model (ADISS AIM) and netCDF software toolkit. The American Society for Testing and Materials (ASTM) is doing the same for Nuclear Magnetic Resonance (NMR). These are expected to be ready by the end of this year, and taken over by the AIA sometime later.

This paper covered the ADISS Technical Architecture and software tools for industry-wide laboratory data standards, and its recent implementations and applications by the AIA, ASTM, LASF, and others. It also covered what work remains to be finished before the standards are fully compliant with Good Laboratory Practices (GLPs) and Good Automated Laboratory Practices (GALPs), which they must address before they are used very widely throughout pharmaceutical, chemical, and environmental testing industries.

### Advancing the state of automated liquid analysis in FTIR spectroscopy

*Michael Garry, Nicolet Instrument Corp., 5225 Verona Rd., Madison, WI 53711 and Cindy Baulsir, Spectra-Tech Incorporated, 2 Research Drive, Shelton, CT 06484*

Fourier transform infra-red (FTIR) technology has been gaining wide acceptance as a method for quality control analysis for a variety of samples. Much of this acceptance has come through the advancement of sampling accessory technology. With advanced sampling techniques, such as attenuated total reflectance, diffuse reflectance, and infra-red microscopy, the ability for analysts to obtain useful spectra from samples quickly has become a much simpler process. Even with these advanced sampling techniques, the average daily sample analysis using FTIR in many laboratories is only in the range of 2 to 5 samples. This is compared to the average daily sample analysis using techniques like high performance liquid chromatography and gas chromatography in the range of 20 to 50 samples. This order of magnitude difference is due in large part to the inability of the FTIR systems run large numbers of samples automatically and to automatically perform quantitative analysis on these samples, as well as the lack of useful methods to analyse samples in the routine quality control environment.

This presentation focused on the advance of software, sampling, and analytical methodology to enhance the use of FTIR in accomplishing routine analysis of viscous liquid easily. The software uses a Windows based operator interface that is easy to use while at the same time being very flexible for using a variety of methods. Fully and semi-automated sampling systems have been developed that are controlled through the software to make sample introduction simple. Methodology exists for routine analysis of important parameters in lubricating oils and edible oils. This represents a significant advancement in the implementation of application specific analysis using FTIR technology.

### Design and implementation of a computer-networked FTIR laboratory

*Robert C. Williams, The BF Goodrich Company, Research & Development Center, 9921 Brecksville Road, Brecksville, OH 44141*

In 1979, the author's infra-red laboratory contained a single FTIR spectrometer. Over the next 12 years, two additional spectrometers and two data stations were added to accommodate the lab's increased workload. Although all three spectrometers came from the same manufacturer, they represented three different generations of evolutionary development, and all of them utilized proprietary computer architecture for their data systems. During this period, attempts to share common resources and/or to transfer data among the three systems were rudimentary and often cumbersome; indeed, the ability to electronically link the three systems were usually addressed only after the instruments had been purchased.

In late 1992, when the oldest instrument was scheduled for replacement, the ability to electronically share both data and common resources was an explicit part of the new instrument evaluation process. After the replacement instrument has been selected and purchased, IR-lab chemists networked it to a PC-clone data station. Total time required to install hardware and make the network operational was less than half a day. With this network, it is now possible for an analyst at the data station to retrieve a spectrum resident on the FTIR spectrometer's host computer, manipulate and plot the data, and search the result against a library resident on the host computer, without interrupting any pending operations on the FTIR spectrometer or requiring any operator input at the host computer. A different 'network' allows the newer, PC-clone based FTIR and the PC data station to share a high-speed plotter with one of the older, proprietary-computer based spectrometers.

The laboratory's early networking efforts were outlined in brief. Network considerations involved in the most recent instrument acquisition were described, and specific design strategy and details of implementation of the current network were presented. Finally, some important networking caveats were discussed.

### Automated determination of total iodine in urine

*T. Nomura, Bran+Luebbe KK, 15–17 Nishi-Shinjuku 8-Chome Shinjuku-ku Tokyo 160, Japan and Stephen Coverly, Bran+Luebbe, Werkstrasse 4, 22844 Norderstedt, Germany*

Hypothyroidism may occur as a result of exposure to radioactivity, dietary iodine deficiency, hormonal disorders and other causes. To help to distinguish between various causes of hypothyroidism it is necessary to check the iodine intake; urinary iodine excretion is the most valid index.

HPLC methods commonly use a pre-treatment with solid-phase extraction, after which unbound iodine is measured. Measuring protein-bound iodine requires a pre-digestion. A previous automated method on the AutoAnalyzer used a continuous Kjeldahl-type digestion, but this needed to be run in a fume cupboard. A method which can be coupled to an AutoAnalyzer has been developed; it performs the digestion on-line in a sealed UV unit, without needing concentrated acid. Following the digestion the liberated iodine is measured automatically. The sampling rate is 30 per hour.

Data were presented on the correlation between the acid digestion and UV digestion methods, recovery and reproducibility.

### Automated sample preparation of consumer products for HPLC analysis

*Susan Kirby Friedman, Cindy A. Mleziva and Bruce P. McPherson, Colgate-Palmolive Company, 909 River Road, P.O. Box 1343, Piscataway, NJ 08855*

Automated sample preparation has seen extensive use in the pharmaceutical industry for solid dosage form testing. The authors' interest is in its use for non-traditional matrices such as mouthrinses, dishwashing liquids and dental creams where sample viscosity and foaming can present significant challenges.

The equipment used consisted of a Zymark Benchmate II Laboratory Workstation with direct injection into a reversed-phase HPLC system equipped with UV detection. Because methodology had to be transferred to other sites worldwide, a robotic workstation was selected over a larger robotic system for its ease of use and transferability as well as its lower cost and wide availability. Although the workstation does offer some flexibility, it has limited functionality and as a result, method transfer issues such as the scaling down of sample size and corresponding dilutions and the conversion from weight/volume to weight/weight quantitation were encountered and resolved. Also, matrix influences such as viscosity in dishwashing liquids and dental creams, and foaming in mouthrinse and dishwashing liquids were overcome.

The laboratory workstation can provide a valuable alternative to manual HPLC sample preparation for a variety of sample types. Data were presented showing a comparison of manual versus automated sample preparations and strategies for dealing with non-traditional matrices were discussed.

### Robotic sample preparation for ICP measurements

*Carmen Piemonti, Luis M. Rivas and Simon Gonzalez, Analysis and Evaluation Department, INTEVEP, S.A., P.O. Box 76343, Caracas 1070A, Venezuela*

A computer procedure, controlling a Zymark robot, was developed and tested to prepare samples and standards for the determination of additive metals in lube-oil by ICP.

ICP readings (I) are recorded for the analyte (A) and the internal standard (La). Both values are associated to the analyte concentration by the following relationship:

$$\frac{I_A}{I_{La}} = B + m[A]$$

The individual readings vary with viscosity and fluid rate changes; however, these circumstances will not affect their ratio. At a constant internal standard concentration, B and m can be evaluated from calibration with standards at different analyte concentrations and regression analysis using the experimental ratios.

The goal of the robotic procedure was to prepare calibration solutions and diluted unknown samples with a fixed lanthanum concentration. In practice, if a concentration of 5 ppm is specified, the prepared samples fulfil this requirement within 0.01 ppm fluctuation.

For both, calibration standards and samples, the analyte dilution factor is also accurately determined. The dilution is necessary to bracket the working concentration range for the metal, however, high precision at this point is not required.

With standards prepared by the robotic procedure, errors on the calibration curves for Ca, Mg, Zn, Ba and Cu can be favourably compared with the manual mode. The effectiveness is also tested with unknown samples by the ICP determination of analytes in the diluted specimens. Precision ranges from 5% (for Ca) to 7% (for Zn), and the accuracy ranges from 3% (for Mg) to 6% (for Cu).

The robotic sequence also aims for minimum material disposal, homogeneity in sample treatments, minimum operator intervention and multiple simultaneous preparation. Some planning is required to: (1) assure availability of reagents, (2) select the proper tool according to the amount of material to be handled, (3) condition the original internal standard concentrates and (4) define analyte concentration in standards. User data analysis focuses on these subjects at the initial step and generates a preliminary report which includes totals for reagents consumption and disposable materials. If errors are detected, warning messages are displayed and corrections by user are allowed, whenever possible, within the same run.

### Development of an automated multi-cylinder inert gas analysis system

*John S. McWilliams and Jeremiah D. Hogan, Texas Instruments, Inc., P.O. Box 650311 M/S 301, Dallas, TX 75265*

Determination of trace levels of contaminants in semiconductor process gases has become increasingly important as device geometries continue to decrease in size. As a consequence, the demand for routine, high-quality analytical techniques to provide the detection limits necessary for such determinations has increased concurrently. This, in turn, has led to increased levels of laboratory automation in all phases of the analytical process, from sample preparation to data handling.

Many types of laboratory samples lend themselves to the automation of the step used in their preparation and/or analysis. Cylinder gas samples, however, present a different set of challenges due to their very nature. Two main obstacles are encountered in the automation of cylinder gases. The first is obvious: the size of the sample container itself. High-pressure gas cylinders weigh up to 300 pounds. The second difficulty is directly related to the first: it is impossible to remove the sample from its container without introducing contamination. Any automation scheme for cylinder gas analysis must address these two fundamental problems.

This work demonstrated the development of a system to allow the unattended sampling and analysis of up to eight cylinder gas samples. A customized cylinder mounting system facilitates the connection of gas cylinders to a manifold leading to the analytical instrumentation. Customized software for controlling the analysis sequence and the methods by which data is collected and process were presented.

### Analytical instrument integration client/server design

*Marc A. Tischler, Hewlett-Packard, Analytical Products Group, 1601 California Avenue, Palo Alto, CA 94304 and Richard B. Opsal, Teknivent, 149 Weldon Parkway, Suite 115, Maryland Heights, MO 63043*

Laboratory automation requires the integration of information acquired from multiple sources. Since analytical laboratories are heterogeneous environments comprised of client instruments from numerous vendors, a centralized laboratory server must be designed with an open architecture that allows clients to pass both data and sample information.

This presentation discussed the Shared File System (SFS) approach used to integrate Teknivent controllers with an HP ChemServer. The term 'SFS' means a file system that is shared among networked computers using a protocol such as Network File System (NFS).

The SFS approach provides several advantages. By acquiring data files directly to the server, the sample throughput improves because the additional step of copying data files is avoided. In addition, regulatory compliance is enhanced because only one copy of the raw data is created. Furthermore, when data files are centralized on the secure file system, Good Laboratory Practices, consistent data processing, disk space management, and backups are simplified. Finally, the SFS approach allows multiple users, with the proper permissions, to access the same copy of the data over the network.

### A new approach to mixing techniques for enhanced performance in automated sample preparation

*Francois Qian, Eric Verette and Atika El-Sayed, Gilson Medical Electronics (France) S.A., 72, rue Gambetta, B.P. 45, Villiers-le-bel, France*

In the automation of sample dilution or derivatization, the performance of the mixing technique employed when adding solvents or reagents to samples is critical. This paper presented a newly developed mixing method, based on conventional aspiration and dispensing of liquid techniques, but which considerably improved the precision of mixing.

A comparison was made of the technique with other mixing methods, such as mixing by air, by mechanical shaking or by ultrasonic means.

The application of the technique to several different types of sample solutions, including a highly viscous glucose solution, were demonstrated. This new mixing technique was performed on a Gilson XL Sampling Injector, with a 1:25 dilution of a paraben solution in 2 ml vials to give relative standard deviations of 0.2 to 0.3% ($N = 10$).

### Continuous hazardous air pollutant emissions monitoring at a pulp and paper mill: system design, function, and data analysis

*Tim Brice, Michael L. Duffy and Gary S. Sides, OI Analytical, P.O. Box 9010, College Station, TX 77482-9010*

With increased pressure to report hazardous air pollutant (HAP) emissions under Title V of the Clean Air Act Amendments and the proposed Enhanced Monitoring Protocol, industries are being forced to assume more of the testing responsibilities in-house rather than through outside consultants. The pulp and paper industry is greatly affected by this legislation.

This presentation described a gas chromatography-based analytical system using a tandem flame-ionization/flame photometric detector to perform analyses based on EPA Method 18. This system extracts a sample from a vent or stack; dilutes the sample with dry air; measures for HAPs, reduced sulphur gases, and other volatile organic compounds (VOCs); and quantifies them continuously. The major system components were illustrated with a discussion of the interferences, data acquisition, and analytical methodology involved at several different sources at one facility. Graphical data showing concentration trends over time at a number of these sources, and maximum and minimum concentrations were presented. This data illustrated how continuous analysers can aid companies in compliance and permitting analyses.

### Monitoring trace contaminants with an automated air analyser

*Mahmood Toofan, Keith Friedman and John Stillian, Dionex Corporation, 1228 Titan Way, Sunnyvale, CA 94088-3603*

Involvement of analytical chemists in atmospheric analysis is significant and growing. This increasing interest calls for improved instruments. Performance goals for these instruments include: chromatographic analysis capable of simultaneous detection of multiple analytes, applicability to acid and base-forming gas samples, sensitivity from mg/litre (ppm) to µg/litre (ppb) concentrations, on-line analysis, and robust reliable operation. Typical applications are: monitoring of acid gases in ambient air, analysis of clean room air for process improvement, and fundamental investigation of air pollution.

The authors' approach to air analysis involves absorbing multiple analytes from the air into a trapping solution, and analysing the solution chromatographically. This presentation described research on a gas/liquid contactor and trapping solution chemistry which optimizes detection for a wide range of analytes. Evaluation and selection of optimum chromatographic methods were discussed. Instrument design for reliable continuous operation was described. Results indicating accuracy, precision and detection limits were presented.

### A continuous emission monitor for incinerators: advanced field test results

*Jack G. Demirgian, Zhuoxiong Mao and Christopher J. Chapo, Argonne National Laboratory, 9700 South Cass Avenue, Argonne, IL 60439*

The public is concerned about emission of hazardous organic compounds from incinerators. This concern is reflected in the USA's Clean Air Act of 1990, which requires air toxics to be monitored from sources that include incinerators. Stack effluents will have to be tested for these compounds by continuous emission monitors (CEMs) when the provisions of the clean air act become effective. Fourier transform infra-red (FTIR) spectrometry can provide the technology for continuous emission monitoring of stacks. Stack effluents can be extracted and analysed in under one minute with conventional FTIR spectrometers. Thus, CEMs would demonstrate the control of air pollutants from incinerators and address public concern. The authors discussed an FTIR system that has been field tested at a Toxic Substances Control Act (TSCA) incinerator at K-25 in Oak Ridge, TN.

Fourier transform infra-red spectroscopy is a promising technology for identifying and quantifying incinerator emissions. This technology takes advantage of the high sensitivity and selectivity of infra-red absorption bands in the fingerprint region of the atmospheric transmission windows. Currently, there is no on-line method that can be used to simultaneously monitor carbon monoxide, methane, and other products of incomplete combustion such as ethylene and benzene. An on-line CEM, however, would demonstrate the safety and reliability of incinerators. The instrument would differentiate species in the parts-per-million (ppm) and parts-per-billion (ppb) range, and in some cases, calculate the destruction and removal efficiency. This information can be used to inform the public about incinerator safety and to smooth the process of obtaining permits.

The authors discussed advances that have been made in developing an FTIR CEM for incinerators. The Environmental Protection Agency (EPA) protocol has been converted into a procedure, which if followed, will provide data quality acceptable to the EPA. The first fully automated, compact, and transportable system has been constructed and tested. The detection levels and analytical results were discussed, and the potential for the technology was evaluated.

### Continuous emission monitoring by an extractive FTIR system. Part I: Performance and verification testing

*Henry Buijs, Daniel Gravel, Allan Rilling and Sam Perry, Bomem/Hartmann & Braun, 450 St-Jean-Baptiste Avenue, Quebec, Canada, G2E 5S5*

An extractive continuous emission monitor (CEM) was described which meets the needs for current pollutant monitoring as well as possessing flexibility for future monitoring requirements. The extractive FTIR based system provides the continuous monitoring of typical components, such as $SO_2$, $NO_2$, NO, $CO_2$, and CO, as well as other components of interest such as HCl, $NH_3$, and HF. The extraction of sample effluent is fully heated thereby maintaining sample integrity and ensuring true sample analysis. Sample analysis is made in the presence of water vapour at levels up to 3% by volume while achieving sensitivities typically less than 1 ppm.

The FTIR based CEM system has undergone a range of testing in an effort to define system performance to be suitable for requirements as defined by EPA's CFR Part 60. The performance characteristics and measuring ranges for each component include a definition of the following; sensitivity, linearity of measurement, level of expected interference, level of drifting, and system response times. The achievable performance as derived for the system was presented along with procedural detail and resulting specifications. The performance verification of performance was also presented.

### Near real-time stack monitoring by in-stack and extractive FTIR techniques

*Richard T. Packard and Ron Shannon, Schuller International, Mountain Technical Center, P.O. Box 625005, Littleton, CO 80162*

The ability to monitor gaseous molecular species contained in industrial emission sources in real time or near real time is key to correlating process parameters to emission characteristics. Fourier transform infra-red spectroscopy provides an effective means for supplying quantitative information regarding a large number of molecular species on a time scale much shorter than that of most process fluctuations. However, there exists some concern that extractive sampling of stack gases into a multipass FTIR cell cannot adequately monitor attack species and events due to adverse effects such as transfer line losses, condensation, or sample decomposition. In addition, fence-line monitoring with open-path FTIR systems is subject to variable weather conditions and a variety of modeling assumptions.

In order to better understand the effects of extractive FTIR sampling, and to provide information necessary to support real-time process variable experiments, an FTIR instrument capable of both in-situ and extractive measurement was used to monitor emission sources associated with fibreglass production facilities. This instrument was designed as a portable unit and is capable of monitoring multiple stacks through heated transfer lines. Results of the comparison between in-situ versus extractive techniques, showing a direct correlation for a variety of stack components such as ammonia, formaldehyde, and methanol were presented. The utility of monitoring several species and sources simultaneously was demonstrated. In addition, the applicability of such an instrument to semi-continuous monitoring of full-scale process variable trials for environmental compliance issues was discussed.

### Development of signal-processing techniques applicable to the monitoring of benzene as an atmospheric pollutant via open path Fourier Transform Infra-red (FTIR) spectroscopy

*D. A. McCarthy, S. A. Dyer, Department of Electrical and Computer Engineering, Kansas State University, Manhattan, KS 66502; R. M. Hammaker, W. G. Fateley, Department of Chemistry, Kansas State University, Manhattan, KS 66502; C. T. Chaffin and T. L. Marshall, AeroSurvey, Inc. P.O. Box 1163, Manhattan, KS 66502*

Environmental monitoring technologies have been receiving increased attention in recent years. One especially promising technique for monitoring atmospheric pollutants at low levels uses infra-red spectroscopic methods to identify and measure airborne compounds. This method is referred to as open path Fourier Transform Infra-red (FTIR) Spectroscopy. Applying signal processing and pattern recognition techniques allows relevant information about a compound to be extracted from a signal. Improved detection capabilities can be achieved in near-real time for a single compound of interest, which in this study was benzene.

Techniques that will produce reliable automated detection of benzene without the need for user input are being investigated by the authors. These techniques will allow for quick and easy resolution of benzene from a spectrum. In addition, these techniques can be applied to the extrapolation of spectral information from other compounds. The results were presented and discussed.

### Enhanced FTIR for multiple-stream environmental air/water monitoring

*D. K. Walker, Marshall University, Dept. of Computer Science, Hungtington, WV 25755; W. P. King, R. B. Lacount, Waynesburg College, Dept. of Chemistry and ViRoLac Industries, Waynesburg, PA 15370; D. G. Kern, R. B. Lacount, Jr. and T. J. Schroyer, ViRoLac Industries, Waynesburg, PA 15370*

This paper described further development of an enhanced FTIR spectrometer capable of multiple-stream environmental air and water monitoring. The system utilizes a Bomem FTIR spectrometer equipped with the recently developed multiple-sample cell positioning accessory, *Enhancer*. The controlling software runs on top of GRAMS/386(Galactic Industries), a Microsoft Windows-based spectroscopy package. Multiple-stream capability is achieved by flowing each sample stream through a separate liquid or gas cell mounted on a linear stepper-motor driven sample table, positioning each cell into the infrared beam at the proper time. Stream switching and cell clearing delays are not encountered with this approach. With new software, up to five different streams per minute may be monitored, along with spectral analysis and updating of on-the-fly display curves showing concentration versus time for each extracted species. Spectra may be re-analysed for the presence of additional species while the original monitoring continues, and the ongoing monitoring and display curves may be modified to include them. Applications for this system include emergency industrial monitoring involving accidental contaminants and chemical release into the air and water, and multiple-stream laboratory applications.

### Improving the analysis of pesticides by optimizing splitless injection

*Philip L. Wylie, W. Dale Snyder, Robert Henderson, Edwin E. Wikfors and T. K. Wang, Hewlett-Packard Co., 2850 Centerville Rd., Wilmington, DE 19808*

The gas chromatographic analysis of pesticide residues is challenging because these compounds are found at trace levels in all kinds of matrices. Pesticides are usually polar compounds which easily adsorb on active surfaces and may decompose when exposed to high temperatures.

Most GC analysis of pesticides is done using splitless injection because it best tolerates dirty samples. However, recent reports have discussed serious problems with this injection technique. In one paper it was reported that sample was being lost out of the septum purge vent of the split/splitless inlet which led to poor recovery values. In another report the authors found that sample matrix was required to deactivate inlet surfaces in order to fully recover certain difficult pesticides. Standards prepared in a solvent without the presence of matrix were subject to adsorption in the inlet causing analyte loss.

This paper explored ways of minimizing losses in the GC inlet by optimizing the splitless injection technique. A unique split/splitless inlet design makes possible dramatic improvements in the analysis of difficult pesticides, leading to much more accurate determinations. The discussion included ways to minimize sample loss out of the septum purge vent, reduce adsorptive losses, and decrease thermal decomposition in the inlet.

### Process control in petroleum industry by Raman spectroscopy

*Ph Marteau[1], N. Zanier[2], F. Cansell[1], A. Aoufi[1], G. Hotier[2] and E. Da Silva[3], 1. Laboratoire d'Ingénièrie des Matériaux et des Hautes Pressions, CNRS, Institut Galilée, Av. J. B. Clément, 93430 Villetaneuse, France; 2. Institut Français du Pétrole, 1 à 4 Av. Bois Préau, 92500 Rueil-Malmaison, France; 3. DILOR, 244ter rue des Bois Blancs, 59000 Lille, France*

This paper described the Raman tool presently used for the control of a paraxylene separation process based upon simulated moving bed chromatography. The spectrometer and the laser are remote with 200 m optical fibres from the sensors located at four different points of the process. These sensors are 'Super Dilor' heads operating through sapphire windows without any generation of the Raman spectrum of silica whatever the length of the fibre. The four Raman spectra are recorded simultaneously on a CCD detector. The relative concentrations of the xylene isomers, of ethylbenzene and of paradiethylbenzene or toluene currently used as the solvent, are delivered every 15 s through quantification and visualization softwares registered under the trademarks SEPAROM and VISAROM, respectively. The real-time Raman system allows the internal concentration profiles to be followed as well as the purity of paraxylene in the extract stream and the loss of paraxylene in the raffinate stream. So, the process can be controlled and optimized. The efficiency of this Raman device has been demonstrated by working 24 hours a day during several months on the Eluxyl® pilot unit in France. The quantication uncertainty referred to chromatographic analyses is of $\pm 0.5\%$ for all values in the 0–100% relative concentration range and $25°–180°$ temperature range with a low fluorescence level. Details of the hardware and of the principle of quantification were given and problems due to fluorescence were discussed.

### *In situ* analysis of thin layer chromatography fractions—a new, simple method for surface enhanced Raman spectroscopy

*Jeremy J. Andrew, Brian P. Newby and Chris J. Adams, Unilever Research, Port Sunlight Laboratory, Quarry Road East, Bebington, Wirral L63 3JW, UK*

Raman spectroscopy has been applied to the problem of analysing organic materials on thin layer chromatography plates. *In situ* analysis avoids time consuming and complex methods which involve the removal of substances from plates. It also removes the risk of contamination during the isolation procedure.

This paper described a new approach to producing SERS activity in order to enhance detection limits in TLC-Raman experiments. Standard silica TLC plates are coated with silver using a vacuum evaporator, the innate surface roughness of the plates being of the correct order to promote SERS activity, if the silver layer thickness is controlled. This approach is superior to methods previously used, involving the spraying of plates with preprepared silver colloids. The method is simple and highly reproducible and the authors' experiments show improvements in limits of detection using this method of over 50 compared to silver colloid spraying.

Using an FT-Raman system, limits of detection in favourable cases are in the low ng levels. These values are equivalent to less than 0.03 $\mu g\ mm^{-2}$ sample loadings. With a CCD-microprobe system at 633 nm, detection limits of subnanogram quantities are easily achieved.

Although not all molecules give good SERS spectra and SERS spectra are not identical to normal unenhanced Raman spectra, which makes identification more difficult, the simplicity and reproducibility of this approach implies that it can be considered for routine analytical application.

### FTIR process monitoring for optimization of $NO_x$ control at coal-fired power plants

*Anthony S. Bonanno, Chad M. Nelson, Kim S. Knight, Michael A. Serio, and Peter R. Solomon, Advanced Fuel Research, 87 Church Street, East Hartford, CT 06108*

Recent legislation in the US and abroad, driven primarily by environmental concerns, has led to the development of technology to reduce the levels of nitrogen oxides ($NO_x$) emitted from combustion facilities. Application of this technology to coal-fired power generation facilities has been particularly important due to the relatively high concentration of nitrogen in coal (0.5–2%). While low-$NO_x$ combustion strategies and post-combustion flue gas treatment can dramatically reduce $NO_x$ levels, it is important to ensure that this is not done at the expense of decreased combustion efficiency or increased levels of other pollutant species. Low-$NO_x$ combustion strategies, such as air staging, reburning, and low $NO_x$ burners, limit the amount of excess oxygen available in the initial combustion stage in order to prevent $NO_x$ formation. Flue gas treatment processes, such as selective catalytic and non-catalytic reduction (SCR and SNCR), employ a reducing agent (usually ammonia) to convert $NO_x$ into molecular nitrogen and water. Poor control of low-$NO_x$ combustion can lead to reduced combustion efficiency and increased levels of unburned carbon, carbon monoxide, and other products of incomplete combustion (PICs). Poor control of SCR and SNCR can lead to increased levels of process byproducts such as $NH_3$ and $N_2O$. A particular problem for both low-$NO_x$ combustion and flue gas treatment is the inability to respond to load fluctuations imposed by boiler demand. In order to effectively implement $NO_x$ control technology at coal-fired power plants, monitoring instrumentation must be expanded to include measurements of species such as $NH_3$ and unburned carbon, and response times must be reduced to satisfy process control demands. *In situ* FTIR emission spectrometry provides a rapid method for monitoring the carbon content in fly ash, without the disadvantages associated with extractive ash sampling. *In-situ* FTIR transmission spectrometry provides the capability to rapidly monitor concentrations of $NH_3$ and other species which are difficult to measure accurately with extractive single-gas monitors. This paper discussed the development and testing of FTIR technology for monitoring parameters of importance to $NO_x$ control processess, and addressed the implementation of this instrumentation in process control strategies.

### Real-time monitoring of metal powder size and temperature by FT-IR spectroscopy for intelligent process control

*Peter Rosenthal, Joe Cosgrove, John Haigis, James R. Markham, and Stuart Farquharson, Advanced Fuel Research, Inc., 87 Church Street, East Hartford, CT 06108; Peter R. Solomon, On-Line Technologies, Inc., 87 Church Street, East Hartford, CT 06108; Stephen Ridder and Frank Biancaniello, National Institute of Standards and Technology, Gaithersburg, MD 20899*

Powdered metals and/or metal alloys are being developed as a source material for a variety of metal forming processes, such as plasma spraying, sintering and hot isostatic pressing. Currently, most metal powder generating systems employ feed/flow control with temperature monitoring of the feedstock crucible and post production sieving for quality control of the particle size distribution. AFR has developed a novel FT-IR system capable of *in-situ* measurements of powder temperature and particle size in a metal powder production reactor. The system employs simultaneous measurement of particle infrared emission and transmission through fibre optic probes to determine temperature, while particle size and distribution is calculated via fast computer algorithms from the transmission spectrum. Real-time data analysis provides the potential for intelligent feed-back reactor control. Integration of the FT-IR system to NIST's supersonic inert gas metal atomization reactor and results of ongoing experiments were presented.

### A novel approach to open path FT-IR spectroscopy

*Robert L. Richardson and Peter R. Griffiths, Department of Chemistry, University of Idaho, Moscow, ID 83844-2343*

The 1990 Clean Air Act Amendments in the USA require documented reduction of 90% of almost 200 air toxics. Many are fugitive emissions and hence are difficult to document using conventional canister analysis. Optical remote sensing can establish an automated round-the-clock perimeter monitoring system. Using a broadband technique such as open path (OP) FT-IR, dozens of compounds can be simultaneously quantified in real time. A major factor currently hindering widespread use of OP FT-IR is the prohibitive cost of the hardware of commercially available systems. The authors have developed an inexpensive, versatile monostatic system based on a fundamentally new approach to atmospheric analysis.

All current OP FT-IR systems use expensive MCT detectors which operate at liquid nitrogen temperatures. This requires either maintaining a supply of liquid nitrogen in the field or use of an expensive microcooler. The authors' spectrometer employs a DTGS detection system not limited by these constraints. In addition, optical hardware for producing and reflecting the collimated infra-red beam comprises a major portion of the cost of current systems, as well as adding considerable size and weight. The system utilizes an optical configuration that is considerably smaller, lighter, and less expensive than conventional ones, resulting in increased efficiency over conventional designs for the same size. In addition, a new type of retroreflector that is superior in performance and cost over conventional cube-corner models has been developed.

The most fundamental departure from conventional design is the use of low resolution (8 to 16 $cm^{-1}$), as compared to the 0.5 to 1.0 $cm^{-1}$ currently employed. In general, the optimum combination of sensitivity and selectivity is found when the resolution is equal to the average full-width at half height (FWHH) of the analytical band of interest. The FWHH of many bands in the vapor-phase spectra of molecules of medium size (about a molecular weight of 100 or greater), such as chlorinated organic solvents, in approximately 20 $cm^{-1}$, so that a resolution of 16 $cm^{-1}$ is often found to yield the most accurate analytical results.

### Minimizing downtime and service costs via remote, modem-based troubleshooting of an automated SFE system

*Mark D. Hansen, Isco, Inc., Separation Instruments Division, 4700 Superior, Lincoln, NE 68504*

As more complex computer-controlled instruments are installed, faster and more efficient ways to service and update instruments must be developed. This is particularly true of instruments used in QA labs at manufacturing facilities that are remote from normal service centres.

Downtime and service costs become critical issues at such remote sites, which are dependent on instrument reliability.

This paper discussed several features in a new automated supercritical fluid extraction (SFE) system which allows remote diagnosis of system problems and rapid updating of the instrument's internal software. Two types of interaction between the remote instrument and service centre were described. One type of interaction uses an MS-DOS-compatible diskette drive built into the instrument. The drive is used to load new software sent via modem to a separate PC at the user's site, without interrupting operation of the instrument itself. This diskette drive is also used to collect diagnostic data which can be transferred to the separate PC and sent to the service centre.

The second type of interaction uses a modem connected directly to the SFE instrument (SFX 3560) to allow data gathering and remote control by a factory-based service technician. This affords the technician a wide range of interactive troubleshooting and fault-correction tools while eliminating travel delays and costs.

Other aspects of system design that enhance remote troubleshooting were discussed, and experiences from beta-site field tests and service department contacts with users were presented.

### Automated fluoride analysis for gases, liquids and solids

*B. C. Kibler, Antek Instruments Inc., 300 Bammel Westfield Road, Houston, TX 77090*

A method and apparatus were described for the automated analysis of fluoride in several different matrices and at several wide concentration ranges. The system described for both laboratory and on-line applications utilizes unique combinations of common technologies to provide matrix independent analysis. Pyro-hydrolysis with ion specific electrode detection and select chemistries are the basis for this innovative approach. Detection limits, linearity, repeatability and reproducibility were discussed for a diverse type of samples, for example, butane, propanes, ethylene, iso-butane, xylenes, paraffins, oils, water, polyethylene, carpeting, biological tissues. Analysis time for laboratory systems of less than 10 minutes per sample was achieved. Range of detection from 0.05 ppm to 70% were presented.

### CAALS-I: a communication specification for instrument-to-controller messaging

*Gary W. Kramer, National Institute of Standards and Technology, Chemical Science and Technology Laboratory, Analytical Chemistry Division, Building 222, M/S B-208, Gaithersburg, MD 20899*

The Consortium on Automated Analytical Laboratory Systems (CAALS), hosted by the National Institute of Standards and Technology (NIST), is a joint venture involving private sector companies and US Government agencies. The Consortium was formed to promote the development of automation in analytical and clinical chemistry, to facilitate the development of standards for laboratory automation, and to develop an environment for making chemical measurement system components interoperable.

CAALS has undertaken, with guidance from its members and others in the analytical instrumentation community, the task of identifying, defining, and promoting general guidelines and standards in critical areas of sample, data, and control information interchange for analytical and clinical chemistry instruments. The Consortium has developed a protocol and syntax specification for communications between instruments and controllers (CAALS-I). This specification, built from existing standards and common practices, is independent of computing platform and provides for guaranteed message delivery and connectivity across several common physical links.

To make it easy for instrument developers, manufacturers, and system integrators to use the CAALS-I specification, the Consortium has developed a number of ancillary materials including demonstration applications on several computing platforms and a verification and conformance testing tool for the module (instrument) end. Currently, a generic code kernel containing a cross-platform-portable library of routines that implement CAALS-I and an implementor's guide are being prepared. This presentation described progress and detailed the availability of these materials.

### Quality assurance (QA) of chromatography data by networked LIMS system

*Kari Aaljoki, Neste Engineering, P.O. Box 310, SF-06101 Porvoo, Finland*

Statistical Process Control (SPC) of analytical results is well established in improving process performance through increased reliability of analytical data in process control laboratories.

This paper demonstrated that easy to use automated standard SPC procedures as well as basic trending, viewing and statistical tools considerably improve the maintenance of chromatographic systems.

The chromatography QA/SPC software described includes the following features:

(1) Automated data capture from a networked chromatography system.
(2) Production of interactive or batch processed SPC charts and regular trends.
(3) Alarming in case of excessive drifts or deviations in (total) peak areas or retention times of quality assurance samples or of process samples.
(4) Very smart automatic scheduling and creating laboratory backlogs of recalibration of chromatographs and of simple maintenance actions such as septum changes.

Analysis of GC history data indicates that repeatabilities of analytical results have improved by about 20% during the first half year of use. It has also helped in tracking down potential problems such as septum or other leaks in GC or sample handling systems, and increased detector noise.

### The original differential spectrophotometry mode in flow injection analysis

*Vladimir V. Kuznetsov and Dong Du Ding, Department of Analytical Chemistry, Menedeleyev University of Chemical Technology of Russia, 9 Miusskaya Square, Moscow 125190, Russia*

Flow injection analysis (FIA) is widely used in up-to-date analytical chemistry. Having used the differential spectrophotometry, the authors have tried to improve this method. An original way of developing this variant in FIA is to use metal-ligand buffer solution with small buffer capacity containing complexone Y and buffer metal ion M at the substoixiometric ratios 1.05–1.1. This variant of flow analysis can be employed in conjunction with a stopped FIA mode.

The conjunction of hydrodynamic injection and displacement reactions allows a specific precision differential photometric system to be used as a low concentration spectrophotometric method. The displacement reactions of copper EDTA chelate with some heavy metals in a flow system containing a coloured indicator were studied. The peak's character depends on the ratio of current concentration of metals to be determined towards one in metal buffer solution (pM). Thus, a 'positive' peak on the response curve can be obtained if the concentration of metal to be determined is over the current pM level. A 'negative' peak is registered when the metal's concentratation is less than the pM concentration. The stopped flow mode allows kinetic problems of displacement reaction to be overcome.

### Near-infra-red monitoring of a polymer extrusion process directly in the extruder

*Jeffrey W. Hall and Peter J. Brush, NIR Systems, Inc., 12101 Tech Road, Silver Spring, MD 20904*

The ability to obtain real-time chemical information directly in the chemical or polymer process stream has initiated a rapid growth in the utilization of process NIR spectroscopy. The opportunity to monitor polymer and chemical processes directly in the process by NIR spectroscopy is afforded by the ability to interface an NIR spectrophotometer to the process by the use of fibre optics and probes.

Process NIR analysis of polymerization batch reactions such as polyurethanes has already been performed. The ability to monitor extrusion processes directly in the extruder has been limited by the inability to monitor strongly light scattering polymerization systems using fibre optic/probe designs which can both be interfaced to the extruder and withstand the extreme temperature and pressure requirements.

This presentation described the use of an extruder interface for a process NIR spectrophotometer for monitoring polymer extrusion reactions which interfaces through standard pressure and temperature transducer extrusion ports. This new process configuration provides improved measurement performance for clear to strongly light scattering polymer matrices and can withstand the extreme temperature and pressure conditions.

### Process monitoring of slurry production using near-infra-red spectroscopy

*Peter J. Brush and Frank A. Dethomas, NIR Systems, Inc., 12101 Tech Road, Silver Spring, MD 20904*

Typical analyses of slurry production require a sample to be taken out of the process stream for analysis in a laboratory. Laboratory analysis requires extra time during which the slurry process continues. If laboratory results later indicate that the process components are not at the correct levels, the entire batch must be reworked to correct the problem. The real time process analysis of the process stream is therefore necessary. Some process sampling techniques require the slurry to be filtered in a side stream loop prior to analytical analysis.

Near-infra-red spectrophotometry allows for the direct process monitoring of slurry production. Real time results are obtained in under one minute, allowing for immediate changes to be made in the process. NIR also allows for the slurry to be analysed without a filtration sampling preparation step. This presentation discussed the monitoring of various constituents during the production of a slurry. Depending on the solids content in the slurry, either diffuse reflectance NIR spectroscopy or transmission NIR spectroscopy can be utilized. Results and problems for each of these approaches were presented.

### Monitoring peroxide in reaction processes

*Peter J. Brush and Stephen L. Monfre, NIR Systems, Inc., 12101 Tech Road, Silver Spring, MD 20904*

Peroxide is used in a variety of reactions. Process monitoring of peroxide concentration is important since it directly affects the yield obtained from most reactions. Differences in peroxide concentration can affect the integrity of the catalyst used in the reaction, the temperature at which the reaction can be performed, and the flow rate at which the reaction can be performed in a tubular reactor. Each of these parameters not only affects the yield obtained from the reaction, but also affect the overall production cost of the reaction.

One solution for monitoring peroxide in reactions is near-infra-red (NIR) spectrophotometry. This presentation showed how NIR was incorporated as a reaction monitoring tool for quinone reactions. Discussions focused on the selection of the appropriate sampling interfaces for peroxide measurements, the selection of multiplexing schemes (multiplexed signals or multiplexed sample streams), and the calibration of the analyser for peroxide determination.

### Monitoring halogen production using near-infra-red spectrophotometry

*Stephen L. Monfre and Denise E. Grzybowski, NIR Systems, Incorporated, 12101 Tech Road, Silver Spring, MD 20904*

Upon completion of halogen production, impurities exist which affect the quality of the product. Typical impurities include moisture, hydrocarbons, haloamines, and halogenated hydrocarbons. Although analyses are performed during production, most of these analyses are performed in the laboratory, which typically means the analytical information has arrived after adjustments to the reaction can be made by production personnel.

Near-infra-red (NIR) spectrophotometry was tested as an analysis method for impurities in halogens. Since halogens contain no NIR absorption characteristics, NIR analysis was found to be an ideal measurement tool for the detection of these impurities. This presentation compared results obtained from the primary method of analysis, Fourier-transform infra-red (FTIR) spectroscopy, with the results obtained from NIR analysis. The NIR results were shown to be equivalent to those obtained through FTIR analysis. Included in the discussion were the requirements for moving the analysis from the laboratory to the production environment, such that real-time information can be provided to production personnel for process control purposes.

### Evaluation of an ambient air sampling system for tritium

*Gregory W. Patton and Andrew T. Cooper, Jr., Pacific Northwest Laboratory, Richland, WA 99352*

Air samples for tritium analysis (as HTO) are collected for Hanford Site environmental surveillance using indicating silica gel, which allows for visual observation of breakthrough. Despite using an established method, breakthrough was observed for some field samples; thus, a series of breakthrough tests were conducted on the sampling system in an environmental chamber. Tests involved both visual observations and recovery studies using a tritium standard.

Experimental: For all tests, flows were 1 to 1.5 litre/min with the relative humidity at 30%. Visual tests were conducted as 20°C to 50°C. Spike recovery tests were evaluated at 20°C and 40°C. The test apparatus was an air pump connected in series to an impinger, a primary column (18-cm × 5.9-cm dia.), and a backup column. Flows were measured and controlled with rotameters. Samples were analysed using vacuum distillation followed by liquid-scintillation counting.

Results: The visual results yielded relative breakthrough volumes (air volumes/adsorbent depth; $m^3/cm$) of 0.39 for 20°C, 0.13 for 30°C, 0.14 for 40°C, and 0.072 for 50°C. Average tritium spike recoveries at 20°C were 71% with no observed breakthrough. Mean tritium recoveries at 40°C dropped from 76% for volumes $< 2.8\ m^3$, to 21% for volumes $> 3.7\ m^3$, which were in agreement with the visual observations.

Field samples are collected using three 18-cm columns connected in series (54 cm total) and 9.5 $m^3$ air volumes. Although the current system is adequate at 20°C, 68 cm of adsorbent would be required at 40°C. Mean summer temperature at the site is 25°C; therefore, the system is adequate for most sampling. Because maximum temperatures can approach 40°C, with temperatures inside the sampling hutches near 50°C, it is necessary to reduce the air volumes collected for summertime samples.

### Field-portable supercritical fluid extraction system for chemical warfare-related compounds

*Bob W. Wright, Thomas S. Zemanian, William H. Robins and Richard N. Lee, Chemical Sciences Department, Pacific Northwest Laboratory, Richland, WA 99352*

During on-site Chemical Weapons Convention (CWC) inspections, low levels of indicator compounds may be present on soil, vegetation, surfaces, processing equipment and dust particles. Rapid sample preparation methods that can be readily implemented in the field are required if these materials are to be subjected to chemical analysis during an on-site inspection. Supercritical fluid extraction (SFE) allows organic extracts to be prepared in as little as 10 min, uses very low volumes of solvent, offers some selectivity, and can be implemented in an automated and user-friendly fashion. SFE methodology investigations indicate that 90% extraction efficiencies can usually be achieved for many chemical warfare (CW)-related compounds and model organo-phosphorus/phosphonate compounds from a variety of solid matrices.

A new prototype SFE instrument was developed for the on-site preparation of solid samples for subsequent analysis. The instrument has two parts, each of which satisfy weight and size requirements for portability. The two parts are a generator module that supplies high-pressure carbon dioxide and an instrument briefcase containing extraction cells, flow restriction hardware, equipment for three different methods of collection (liquid solvent, adsorbent, or restrictorless rapid depressurization), control apparatus, and operating supplies.

The design of the prototype field-portable SFE instrument was described, as well as the technical performance of the instrument both in the laboratory and in the field for CW-related compounds such as simulants, model compounds, and degradation products.

### Detecting organic compounds with a transportable FTIR gas analyser

*Erkki Kantolahti, Tarmo Humpii and Tiina Niinimäki, Department of Chemistry, Defence Forces Research Centre, FIN-34111 Lakiala, Finland*

A study has been undertaken to investigate the potential of a transportable FTIR gas analyser for monitoring organic compounds in the air. The Gasmet gas analyser, manufactured by Temet Instruments Oy, Finland, was used as an experimental apparatus. The analyser consists of a FTIR spectrometer, a temperature-controlled gas cell, signal processing electronics and a 486 computer with Calcmet control and analysis software package.

The Gasmet multicomponent gas analyser allows simultaneous measuring of up to 21 gaseous components continuously. The algorithm of the analyser uses library spectra to build a spectrum that matches the unknown spectrum. The reliability of the analysis can be estimated by comparing the difference between the observed and calculated spectrum.

The infra-red region 950–4000 $cm^{-1}$ with 8 $cm^{-1}$ spectral resolution was used in all measurements. The gas cell had a volume of 4.4 l and a pathlength of 9.6 m. The cell temperature was maintained at 50°C. The compounds studied were volatile organic solvents and organophosphonates. The detection limits calculated were 0.03–0.1 ppm. The Gasmet gas analyser was able to reliably and quickly resolve compound studied from the sample, where water, $CO_2$ and some hydrocarbons were present as a background mixture.

### Alternate automatic GC analysis—the solution for on-site temperature ramping

*A. Linenberg and Gene Q. Song, Sentex Systems, Inc., 553 Broad Avenue, Ridgefield, NJ 07657*

EPA Methods for GC analysis of air or water often require temperature ramping. These methods are intended primarily to detect a large variety of chemicals. To perform such an analysis a sophisticated laboratory gas chromatograph is used to assure the proper accuracy and reproducibility in operating the temperature ramping. However, there are many occasions where it is advantageous to perform an on-site analysis using a portable gas chromatograph. Portable gas chromatographs, on the other hand, either do not have this capability or if they do have it, the accuracy and reproducibility of temperature ramping is not very reliable and results may be affected accordingly.

A solution to that situation was found in the development of a portable gas chromatograph system by which two or three columns operate isothermally with sequential sample analysis. In the two-column system, two columns were placed in the same oven operating isothermally and sharing a same detector. The two columns were selected to have a different nature so that they would yield different retention times when used for the same sample. One column was used for light or volatile compounds like vinyl chloride, dichloroethane, etc. The other column was used for heavier compounds like xylenes, chlorobenzene, etc. During the calibration cycle, one column was first used to analyse a calibration standard and to set up the calibration parameters for that sample. After the system finished the analysis using the first column, it switched automatically to the second column to sample from the same calibration standard, and to repeat the same cycle as for the first column. During the analysis cycle, the system analysed a sample using the first column against the first calibration. Sequentially, the system switched to the second column to analyse the same sample against the second calibration. In this manner, while one column can separate the light compounds in a sample, using the same temperature the other column can separate the heavy components in the same sample.

Software for automatic calibration, analysis, and backflush at the end of each analysis was developed to accommodate this system. The system allows relatively fast analysis of both heavy and light compounds isothermally and accurately without the need for temperature ramping.

### New applications for a heated, portable micro gas chromatograph

*Kent G. Hammarstrand and Debra L. Gonzalez, MTI Analytical Instruments, Microsensor Technology, Inc., 41762 Christy Street, Fremont, CA 94538*

A novel heated sample inlet and silicon micromachined injector for a micro gas chromatograph has been developed and applied to various higher temperature gas analysis applications. The application of silicon micromachining technology to the fabrication of analytical instrumentation has yielded a high speed, portable gas chromatograph capable of analysing gas phase compounds which are present in part-per-million level concentrations at standard temperature and pressure. With the addition of a heated sample inlet and injector, the addressable analytical sample range of this gas chromatograph has been extended to higher boiling point/molecular weight samples with minimal sample carryover and greater analytical reproducibility. With the incorporation of the heated inlet and injector, the micromachined gas chromatograph can analyse lower levels of high boiling point compounds (i.e. semivolatile compounds such as naphthalene) and higher levels of compounds that might otherwise condense in the sample inlet and injection system (for example compounds near their saturation vapour pressure concentration).

The fabrication of a heated sample inlet and silicon micromachined injector and their integration into a portable micro gas chromatograph was presented. The extended application range, sensitivity, reliability, accuracy and high reproducibility of this instrument was highlighted relative to an unheated system. Applications of this instrument to environmental and process samples (for example refinery gas analysis) were described.

### Validation of computer systems in the environmental laboratory

*Ludwig Huber and Mike Thomas, Hewlett-Packard GmbH, Hewlett-Packard Str., 76337 Waldbronn, Germany*

While most environmental laboratories are familiar with validation of equipment hardware and analytical methods, the validation of computer systems is not so well understood. However, this is strongly recommended by the Good Automated Laboratory Practice (GALP) guidelines published by the United States EPA.

The poster provided guidance for QA and laboratory managers and users of computer-controlled analytical instruments on the entire validation process, from design through implementation, testing, qualification, to calibration and performance qualification.

The poster covered of most regulations, official recommendations and accreditation standards such as GLP, GALP and ISO Guide 25. The concept, examples and many templates included have evolved from the authors' experience with validation processes as applied in their company and from intensive discussions with regulatory agencies, users of equipment, corporate QA managers, instrument vendors, and consultants in personal interviews and during numerous seminars conducted by the authors.

The poster gave recommendations on how to speed up the validation process and how to do it 'right' first time thus building confidence for audits and inspections and avoiding troublesome rework.

### Automated Soxhlet extraction and extract concentration for determination of oil and grease in sludge and soil

*Kevin P. Kelly and Evelyn E. Conrad, Laboratory Automation, Inc., 555 Vandiver Dr., Columbia, MO 65202*

Spiked prepared dried soil samples and digested municipal sewage sludge samples (acidified) were extracted with *n*-hexane or with Freon 113 (1,1,2-trichlorotrifluoroethane) using a new automated Soxhlet extractor to determine oil and grease content by gravimetric technique. The procedure employed was an adaptation of US EPA methods.

The method yielded accurate results while consuming less preparation and analysis time, labour and solvent than does the traditional Soxhlet procedure. Additionally, the system used automates both the extraction and evaporation portion of the procedure, and recovers spent solvent in a form that allows for easy recycling of the recovered material.

Recoveries near 100% were obtained for sludge samples spiked with a mixture of corn oil and diesel fuel and extracted with Freon. Precision was not as good for sludge samples as it was for soil samples. This was probably due to the effect of matrix inhomogeneity when preparing aliquots of the sludge samples, which contained approximately 5% suspended solids.

Freon used in these experiments was recycled with a spinning band distillation system. Freon used as extraction solvent has been granted an exemption to next year's ban on manufacture and sale, and use of recycled Freon is permitted under terms of the ban, but EPA still proposes elimination of the Freon-based techniques for ecological reasons. Therefore, data were also presented for a hexane-based adaptation of the technique. Like Freon, hexane can be recovered using this technique and recycled.

### Calibration bracketing and automated reporting in pharmaceutical manufacturing

*R. DePinto, Ganes Chemicals, Inc., 611 Broad St., Carlstadt, NJ 07072; K. McDonald, S. Jenkins, Pharmaceutical Associates, P.O. Box 128, Conestee, SC 29636; F. O. Geiser, T. Peterson, G. Schreiner and J. D. Justice, Justice Innovations, Inc., 1240 L'Avenida Ave., Suite A, Mountain View, CA 94043*

Chromatographic assays in pharmaceutical manufacturing typically require compliance with USP system-suitability specifications. Justice Innovations' Chrom Perfect for Windows data acquisition system was developed to be fully USP compliant. A procedure was described that preserves chromatographic data integrity by generating bracketed calibration reports from bound, binary data files. The flexible technique is suitable for use with either external or internal standards.

### On-line derivatization for gas chromatography using an injection port mounted microreactor

*Thomas P. Wampler and Woodford A. Bowe, Jr., CDS Analytical, Inc., 7000 Limestone Road, Oxford, PA 19363-0277*

Pre-chromatography sample treatment continues to extend the range and application of gas chromatography in the analytical laboratory. Derivatization prior to injection improves chromatographic performance for polar and other problematic samples. This paper presented data obtained by converting the sample materials after injection, by passing the analytes through a small reactor placed between the injection port and the analytical column.

Use of a variety of catalysts and reagent gases permits use of the on-line derivatization/conversion reactor in several modes. Samples may be hydrogenated to eliminate unsaturated materials and simplify the resulting chromatogram. Organics may be reduced to methane for a total carbon determination. Further, a combination of oxidation and reduction catalysts permits the conversion of organic carbon, hydrogen and nitrogen to small molecules, permitting the use of a standard gas chromatograph for elemental analysis.

Examples were shown of various modes of operation, including pyrolysis-hydrogenation-GC for simplified polymer analysis, total carbon analysis using methane conversion, and elemental analysis of both solid and liquid samples.

### Development of automated SFE methods for fat determinations

*Athos C. Rosselli, Joseph M. Levy, Victor G. Danielson, Anita Cardamone, Robert M. Ravey and Glenn Muzyk, Suprex Corporation, 125 William Pitt Way, Pittsburgh, PA 15238*

The authors described the steps used to develop a successful SFE method for the quantification of fats and oils from a variety of food and pharmaceutical products, including potato chips, animal feed, soybean flakes, corn chips and infant formula. Current methodology commonly relies on exhaustive extraction with liquid solvents, such as chloroform, hexane, or petroleum ether, in a process which can take up to 12 hours. SFE can greatly reduce this time and eliminate most of the liquid solvents used. This paper addressed the selection of the appropriate extraction temperature, pressure and modifier, with data presented to illustrate the effect of each of the parameters. The optimization of extraction time was discussed, with graphs to indicate the optimum balance between recovery and length of extraction. Data were presented to show the effect of flow rate on both recoveries and extraction duration, and the role of the restrictor, especially the new automatically variable type, was addressed. The relative merits of modifier addition versus higher temperature and pressure was highlighted, and some guidelines for their use detailed. Data showing the repeatability of an optimized extraction were presented with interlaboratory validation.

### Automated determination of total phenols in wine

*Fran Lai, Landy White, Lenore Kelly and Gary Engelhart, Thermo Separation Products, 45757 Northport Loop W., Fremont, CA 94501*

Phenols are major secondary chemicals in red wine. They contribute red pigment, as well as taste to the wine. An excess of total phenols would cause bitterness and insufficient phenols would cause insufficient colour and/or taste. It is therefore very important to ensure the right amount of total phenols in the quality control process.

An existing method for the determination of total phenols is a manual one in which multiple steps are involved in the addition of reagents and diluents. It also requires an incubation period prior to analysis. This requires the operator to be multi-tasking between sample prep and analysis. Uniform and precise timing for all samples could also be a problem.

In this paper the authors presented an automated method using an autosampler with microrobotic capabilities for the whole process including sample prep and analysis. The autosampler is equipped with a holding loop and a 2.5 ml syringe for reagent and diluent addition. It also has a heater/mixer in which mixing can occur at room temperature or elevated temperature. The sample tray is equipped with heating/cooling options for further incubation or sample cooling. The autosampler is controlled by a customized method development language for the syringe and valve movements to facilitate the sample prep and direct sample loading on the detector for spectrophotometric analysis. This robotic system allows multi-tasking between sample prep and analysis and guarantees uniform and precise timing for all samples.

### Automatic internal standard introduction in a 24-sample SFE system

*Phillip B. Liescheski, Dale L. Clay and Earl Bush, Isco, Inc., Separation Instruments, Division, 4700 Superior, Lincoln, NE 68504.*

Internal standards are important for the calibration and control of many analytical methods. With automated SFE using liquid solvent trapping of the extracted analyte, it can be useful to the chemist to automatically introduce an internal into each extract solution before the extracts are analysed. In the automated 24-sample Isco SFX 3560 extractor, which uses liquid trapping of extracts, the collection vials are automatically filled with solvent before extraction, and replenished with new solvent during and after the extraction. During the replenishment step, a precise volume of internal standard can be added to each vial.

The internal standard solution is volumetrically added to the solvent replenishment stream using an HPLC injection-type rotary valve and sample loop. Under program control, the rotary valve is set to 'load' and the sample loop is filled with the standard solution. The rotary valve is then set to connect the filled sample loop in series with the solvent replenishment line. The solvent replenishment pump is activated, and the solvent flushes the internal standard into the collection vial.

Data were presented showing chromatographic reproducibility for extract solutions collected using the automatic internal standard addition. Information on the coupling of automated SFE to HPLC was also included.

### Teaching and re-training using electronic classrooms

*V. Karanassios, Department of Chemistry, University of Waterloo, Waterloo, Ontario, Canada N2L 3G1*

The fast pace of change requires current and former students to keep up-to-date with recent scientific and technical developments. Toward this goal, two electronic classrooms located about 25 km apart have been developed. The two identical classrooms (one at Waterloo and one at Guelph) accommodate 38 students each. Students sit in desks in pairs and share a microphone and a video monitor. The classrooms are linked by symmetrical microwave channels that carry video, audio, data and still images (for example, from an electronic white board, from an overhead projector or from a slide projector) in real-time. The two-way link allows faculty to lecture from either location. The system is fully interactive and students can participate in class discussions.

Recently, MacMaster University has joined the 'link'. Their classroom connects with Guelph using a dedicated fibre optic telephone line and with Waterloo using the microwave link. The classrooms were described and the potential of video-phone and of global dial-up video conferencing systems for re-training and for the development of virtual electronic classrooms were outlined.

### The development of a diffuse reflectance probe for on-line, real time process monitoring and control

*Richard D. Driver, Kirk P. Grim and Monte L. Brubaker, Galileo Electro-Optics Corporation, Sturbridge, MA 01566*

The majority of manufactured materials, including many webs, solid materials and liquid phase materials, are optically inhomogeneous and unsuitable for analysis with conventional absorption spectroscopy. The Diffuse Reflectance Infra-red Technique (DRIFT), offers an alternative sampling method which yields well defined infra-red spectra which are reproducible and may be used for quantification of multi-component materials.

With suitable design parameters such a system may be used for on-line or at-line sampling. A new Diffuse Reflectance System has been developed which allows extremely high signal to noise levels to be realized with most inhomogeneous samples with dispersive, APTF or FTIR spectrometers. The use of diffuse spectroscopy is non-destructive and may be carried out in real time during manufacturing.

Data were presented for the use of diffuse spectroscopy in chemical manufacturing. Derivative spectra of mixtures of polymer additives give accurate information on blending distribution. The diffuse spectra of pharmaceutical tablets give accurate information on tablet hardness on real time. The technique may also be used to analyse and separate construction materials based on safety considerations. Multiplexing schemes for multpoint diffuse spectroscopy systems were discussed.

### Automated tablet processing: sample preparation and HPLC with photodiode array detection

*Patricia M. Young, Raymond P. McGuirk, Waters Chromatography, 34 Maple St., Milford, MA 01757 and James Martin, Source for Automation, 115 Cedar St., N-3, Milford, MA 01757*

Regulatory demands require content uniformity and composite assay testing for most solid dosage forms manufactured in the US. Typically, these tests are done manually with labour spent weighing and diluting samples, dispersing the tablet matrix, and maintaining accurate records. The requirement for high throughput and comprehensive management of the sample preparation process, tablet weights, dilutions, content analysis and data reporting to complete the audit trail for GMP/GLP compliance has made automation essential. All segments of tablet processing, from sample preparation to component quantification, can be accomplished with the Waters Tablet Processing System.

Data from sample preparation are stored in the relational database at the heart of the Waters Millennium Chromatography Manager. While spectrophotometric analysis may be effective for dosage forms having a single component, increasingly, purity assessment and drug stability assays are required. These tests, combined with the need to quantify all ingredients in a multi-component drug, have increased the necessity for HPLC separations. With on-line photodiode array detection, full spectral data regarding tablet content and possible impurities can be obtained and stored in libraries for identification. Thus, WTPS provides a validated platform for automated continent uniformity testing, component quantification and results management.

### An overview of the validation studies for automated procedures for the analysis of NIDA-5 drugs of abuse from urine using an integrated sample preparation/GC/MS system

*Wayne S. Miles, Charles R. Knipe, Patricia L. Castelli, Hewlett Packard Company, Little Falls Site, Wilmington, DE 19808 and Bruce A. Goldberger, University of Florida, College of Medicine, Gainsville, FL 32610*

The increasing demand on laboratories to analyse large numbers of clinical and forensic specimens has drawn their attention to the consideration of automated methods of analysis. For many laboratories one of the tasks that automation presents is the successful conversion of manual methods to automated methods. An example of this approach is the conversion of the methods for sample preparation, confirmation, and reporting of results for parent compounds and metabolites of the NIDA 5 drugs of abuse. In these methods the usual procedure involves sample preparation (adjustment of pH, addition of ISTD, protein precipitation, etc.) followed by isolation of the analyte from the matrix, with subsequent confirmation and data reporting.

In the work reported, matrix isolation was accomplished by an automated solid-phase extraction (SPE) module coupled to a GC/MS system for analysis and confirmation. Data analysis and reporting was handled by a commercial reporting package with format specifically targeted for laboratories involved in analysis of drugs of abuse. Results of assay characterization, accuracy and precision studies, parallel studies with patient specimens, and carryover studies, were presented for cocaine/benzoylecgonine, THC, amphetamines, PCP, and opiates. In all instances overall %CVs were less than 4% and no measurable carryover was observed in the concentration ranges studied.

### On-line supercritical fluid extraction/gas chromatography of environmental samples

*Howard T. Mayfield, Michael V. Henley, Bruce J. Nielsen, Armstrong Laboratory, AL/EQ, 139 Barnes Drive, Suite 2, Tyndall AFB FL 32403-5323 and Larry E. Gerdom, Mobile University, Division of Natural Science, P.O. Box 13220, Mobile, AL 36663-0220*

An instrument combination for performing on-line supercritical fluid extraction/gas chromatography (SFE/GC) has been assembled. In this instrument, the transfer line/restrictor from the supercritical fluid extractor was introduced into a conventional back pressure-regulated split/splitless injection port of the gas chromatograph. In an effort to preserve the integrity of volatile compounds in the extracted samples, the oven of the gas chromatograph was cooled to low subambient temperatures. A 1 µl injection loop, installed upstream of the extraction cell was used to inject internal and quantitation standards for calibration of the system.

In a test of the application of this instrument and technique to practical samples, split soil samples were collected and analysed from several points around a suspected fuel spill site. The soil samples from the spill site were taken with the guidance of a cone penetrometer system equipped with a laser-induced fluorescence sensor (LIF). Results of the analysis of the soil samples were compared with the LIF readings taken during the soil sampling and with the analytical results of the splits using an off-line supercritical fluid extractor of current design and construction, equipped with a cryogenically cooled, packed trap for the collection of extracted components.

### Development of sensor arrays for continuous ground water monitoring

*Brian G. Healey, Suneet Chadha, David R. Walt, Max Tishler Laboratory for Organic Chemistry, Tufts University, Medford, MA 02155, USA; Fred P. Milanovich, James Richards and Steve Brown, Measurement Sciences Group, Physics Department, Lawrence Livermore National Lab, Livermore, CA 94550*

Industrial development has led to the release of numerous hazardous materials into the environment, posing a potential threat to surrounding waters. Environmental analysis of sites contaminated by several chemicals calls for continuous monitoring of multiple analytes. Monitoring is achieved by photodepositing different analyte-sensitive polymer matrices on the distal end of a single imaging fibre. First the distal end of the fibre is cleaned and subsequently functionalized to permit covalent attachment of analyte-sensitive polymer matrices to the fibre. The initiation light is focused onto the proximal end of the fibre in a very discrete region. The distal end of the fibre is then placed in a monomer solution, with photoinitiator and fluorescent indicator, and irradiated for a given time. Polymerization occurs only at the illuminated area of the imaging fibre. After illumination and removal of the residual monomer, the initiation light is focused onto a different region of the fibre. The process is then repeated to give the next analyte-sensitive polymer matrix.

Current studies focus on the development of a fibre optic sensor for monitoring $Al^{3+}$, pH, hydrocarbons and uranyl ion. For the monitoring of $Al^{3+}$, a variety of indicators are being evaluated for their applicability to sensor design. The fluorescent indicators eosin (pH 2.0–4.0) and fluorescein (pH 5.0–7.5) have been immobilized for monitoring pH. Hydrocarbon sensors have been fabricated from different photopolymers which show response to several hydrocarbons using Nile Red as the indicator.

### Automated solutions for gasoline analysis, including ASTM method 3606, aromatics in gasoline and RFG products

*Patricia Castelli, Lenore G. Randall, Chuck Knipe, Hewlett-Packard, Little Falls Site, 2850 Centerville Road, Wilmington, DE 19808 and Diane Lamonica, Analytical Controls, 3448 Progress Drive, Bensalem, PA 19020*

This paper described the automated preparation of calibration standards and gasoline samples for various methods used in the petrochemical industry. Results obtained on automated PrepStation/6890 GC system were presented. ASTM method 3606 using a PrepStation/6890 GC system was shown as one of the examples. ASTM D-3606 is used to determine the percent benzene and toluene in gasoline products.

Results for the preparation of calibration standards showed correlation coefficients when standards were prepared with the PrepStation/6890 GC system. The relative standard deviation for the entire process (preparation and analysis) was also presented, showing the results of 25 gasoline samples.

The automated system has several advantages over more traditional preparation techniques. PrepStation uses less solvent than traditional methods, this results in lower solvent purchase and disposal cost. Less attended operation time results in savings in operator time while reducing operators' exposure to hazardous chemicals. Just-in-time delivery of the sample with this system reduces loss of volatile compounds. Cost savings can be substantial over a period of time. The combination PrepStation/6890 GC provides a very sensitive analytical methodology.

### A new instrument for automated cold vapour analysis of mercury

*Robert Moseley, Thermo Jarrell Ash Corporation, 8E Forge Parkway, Franklin, MA 02038; Robert Ballantyne and Keith Ballantyne, Scientific Measurement Systems, Inc., 606 Foresight Circle, East, Grand Junction, CO 81505*

Instrumental design parameters key to the process of optimum mercury analysis were examined in this study. Data presented showed the impact of optimizing on-line optical feedback upon stability and detection limits. Temperature control of the mercury line source is an important design parameter in control of stability. The thermal design of the optical measurement assembly improves sensitivity and detection limit. The fluid gas interface is designed to optimize drift, carry-over and linearity. The instrument includes a new method for phase separation of water vapour and other potential interferences. The paper also examined amalgamation and automatic sample preparation as a part of the process.

### Digital pressure control and flow feedback for increased performance in an automated SPE workstation

*C. B. Shumate and J. E. Johnson, Hamilton Company, 4970 Energy Way, Reno, NV 89520*

Existing commercial automated SPE workstations operate with either vacuum or positive pressure for conditioning, sample addition, washing, and elution. The advantage of positive pressure comes from increased control of pressures and flow rates. A problem encountered with both approaches comes about from variability in column packing as well as sample viscosity and suspended solids. Columns or samples with significant resistance to flow will often fail to be properly processed and subsequently invalidate an entire run. The use of a proprietary digital pressure/flow transducer has allowed continuously adjustable computer controlled pressure regulation for high flows in the case of washing and drying steps and very low flows for equilibria dependent sample addition and elution. Additionally, the detection of high flow resistance column/sample combinations can be responded to through a variety of contingencies. Pressure can be increased to initiate flow, an organic wetting agent can be added to facilitate flow, or the sample can be directed to the next column. Automatic dilution or other sample pre-treatment is possible. The sample can also be removed from the worklist and the technician notified of its failure. This flow interrogation allows independent processing time for each sample, greatly decreasing total run times resulting from an arbitrarily long pressure pulse being applied. Column drying, and hence low recoveries, are eliminated and precision within runs is greatly increased. The design and use of this system was discussed and data were presented demonstrating the increased performance of the resultant automated SPE workstation.

### The use of automated SFE/GC for the quantitative determination of pesticides in soils and feeds

*Lori A. Dolata, Joseph M. Levy, Robert M. Ravey, Anita Cardamone, Athos C. Rosselli and Kathy Holowczak, Suprex Corporation, 125 William Pitt Way, Pittsburgh, PA 15238*

Supercritical Fluid Extraction (SFE) has evolved to a point where it has gained a significant amount of attention by the scientific community. However, widespread acceptance of SFE cannot be attained unless pertinent applications can be demonstrated. Such applications were presented and discussed with respect to the quantitative determination of various carbamate, organochlorine, and organophosphorous pesticides in selected soil and feed matrices utilizing off-line SFE and GC-mass spectrometric and electron capture determinations. Moreover, an example of the non-trivial extraction of pesticides in fat was discussed, focusing in on the various experimental details that were employed for successful extraction of the pesticides without the interfacing co-extraction of fat related compounds.

### The automation of peptide and protein desalting

*Rick Gard and Andre Szczesniewski, Hitachi Instruments, Incorporated, 3100 N. First Street, San Jose, CA 95134*

Often the time-limiting step in the characterization of proteins and peptides is the time and trouble required to desalt the sample after purification. This presentation described the process of using a programmable robotics autosampler to automate the desalting of protein and peptide solutions prior to analysis by mass spectroscopy or any other technique in which removal of excessive salt is a necessary step. The procedure described in this paper requires some simple hardware modifications of the autosampler, the use of solid phase desalting cartridges, and generation of a custom autosampler program. Up to 50 samples may be desalted in one run, and 20 min are required to desalt each sample. The recovery of protein ranges from about 50% to 100%, depending on the protein. The recovery of peptide is usually over 90%.

Desalting examples were shown to illustrate how the system performs. A complete discussion of the hardware operation and modification was given. Programming is accomplished using a Fortran-like robotics language. An explanation and listing of the autosampler macros that were used was given.

### Automated Soxhlet extraction, cleanup, and concentration for determination of chlorinated pesticides in low-fat meats

*Nancy L. Schwartz, Evelyn E. Conrad and Kevin P. Kelly, Laboratory Automation, Inc., 555 Vandiver Dr., Columbia, MO 65202*

A new automated system for Soxhlet extraction is used to extract low fat meat products. Since the fat extract is less obtainable from meats of lower fat content, the Soxhlet procedure is proposed to produce a less labour intensive, yet thorough extraction method. Extraction occurs in two stages with this procedure. First there is an initial boiling time, during which the sample is immersed in boiling solvent. The second stage is similar to the more traditional Soxhlet-type extraction, wherein the sample is extracted by a continuous refluxing of condensed solvent.

Between these stages and after extraction, solvent volume is automatically reduced by diverting a portion of the condensed solvent vapours before they come in contact with the sample and contribute to the volume of accumulated extract.

Samples were spiked with a mixture of chlorinated pesticides prior to automated Soxhlet extraction. Automated equipment was also used to clean the extract by GPC (gel permeation chromatography), during which the eluate was concentrated on-line as the desired fraction eluted. Recoveries, determined by GC/ECD, were close to 100% with good precision.

The combination of automated extraction and automated cleanup and evaporation shows that samples for fat determination or trace level contaminant quantitation can be processed rapidly and with minimal labour requirement relative to traditional methods.

### Optimizing the role of automation in the pharmaceutical laboratory: three case studies

*Susan G. Kelley, Source for Automation, Milford, MA 01757*

The routine use of automated chemistry workstations has increased in pharmaceutical laboratories over the last few years. Specifically designed for content uniformity and composite assay testing, these workstations are benefiting pharmaceutical QC, method development and stability laboratories. Each type of laboratory demands that automation assumes a different role to increase productivity appropriate to that lab's function. Optimal utilization is achieved for workstations in each setting through careful study of the lab's work flow.

The testing done in these laboratories includes release, stability, process validation, method development, research and development and in-process testing. Case studies demonstrating increased efficiency in several laboratory situations were presented. Included were examples of sample schedules and loading, increased productivity, return on investment and the increased productivity of multiple workstations.

### An on-line interface between microdialysis and capillary zone electrophoresis

*Mark W. Lada (1), Gabrielle Schaller (1), Marie H. Carriger (2), Thomas W. Vickroy (2), and Robert T. Kennedy (1), (1) Department of Chemistry, P.O. Box 117200, University of Florida, Gainesville, FL 32611, (2) Department of Physiological Sciences, 100144 JHMHC, University of Florida, Gainesville, FL 32610*

A flow-gated, on-line interface between a microdialysis sampling probe and capillary zone electrophoresis with UV absorbance detection was characterized and applied. With the system fully automated and using ascorbic acid as a test analyte, it was found that injections could be performed every 50 s. Ascorbic acid migrated with 50 000

to 120 000 theoretical plates depending on the conditions used. Theoretical plates were the same as those obtained for conventional injections of ascorbic acid. These results were obtained using a 25 μm inner diameter capillary with an inlet to detector length of 15 cm and electric field strength of 600 V/cm. The interface allowed injection of samples from the dialysis probe with dialysis flow rates as low as 79 nl/min. This compares to the 1 μl/min flow rates that are commonly used. With low dialysis flow rates used, the relative recovery of the probe (the concentrate of analyte in the dialysate relative to the concentration outside the probe) was 98%. The high relative recoveries improved detection limits, simplified quantification, and resulted in decreased disturbance to the system being studied. The relative standard deviation for peak heights was 2.0% and a linear response over the physiologically relevant range for ascorbic acid was observed. As a demonstration of the system, ascorbic acid in the caudate nucleus of rat brain was detected and monitored in response to amphetamine injections and anesthetic overdoses. This system is the first to allow high relative recoveries and high time resolution simultaneously with microdialysis sampling.

### Automated liquid–liquid extraction of semivolatile analytes from aqueous samples

*Kevin P. Kelly, Evelyn E. Conrad, David L. Stalling and Nancy L. Schwartz, Laboratory Automation, Inc., 555 Vandiver Dr., Columbia, MO 65202*

Traditional methods for extracting analytes from aqueous environmental samples are liquid/liquid extraction in separatory funnels or in glass continuous liquid/liquid extractors. Much time and manual labour is required and the separatory funnel method is often impaired by the formation of intractable emulsions.

A new automated liquid/liquid extractor utilizes a new principle of enhancing the surface–surface contact of the mixed phases. Intimate phase mixing is accomplished through application of an electric field to droplets of aqueous phase as they pass through extraction solvent. Emulsions have not been encountered. All phases of the process are automated, including metering and delivery of extraction solvent, timing of process stages, and rinsing of the extractor at the end of the operation. Built-in sensors detect the organic/aqueous interface and cause the organic and aqueous phases to go to separate destinations. The extracts are automatically collected in convenient glass bottles for easy transfer or storage. The aqueous phase can be (automatically) discarded or retained for further processing, such as a pH change. The instrument processes aqueous samples traditionally extracted with, for example, US EPA SW-846 Methods 3510 and 3520. Up to six samples can be extracted simultaneously. A set of extractions can be completed in under three hours.

Spiked real and simulated samples were extracted and the extracts were subsequently analysed by GC/ECD or GC/FID. Precision and accuracy data are presented. Reagent water spiked with surrogate and matrix spiking compounds (BNA), extracted at $pH<2$, evaporated using nitrogen, and analysed by GC/MS yielded recoveries ranging from 39% (phenol) to 94% (pentachlorophenol) with standard deviation of 1.3 to 7.7 for six replicates. Similarly, pesticide and PCB surrogates and matrix spiking compounds (GC/ECD analysis) gave recoveries of 78% to 106% with standard deviations of 1.1 to 3.5. Principles of extractor operation were discussed.

### Automated Soxhlet extraction of semivolatile analytes from soils

*Evelyn E. Conrad, Kevin P. Kelly and Nancy L. Schwartz, Laboratory Automation, Inc., 555 Vandiver Dr., Columbia, MO 65202*

A truly automatic Soxhlet-type extractor provides unattended two-stage extraction of solid samples (for example soils) for semivolatile compounds often cited as environmental pollutants. In the first stage of extraction, the sample is immersed in a boiling solvent which facilitates extraction of target analytes, thereby shortening the total extraction time required. The boiling solvent level is then lowered automatically by shunting away aliquots of condensed solvent. The second extraction stage is similar to a Soxhlet-type of extraction during which the condensed solvent percolates down through the sample.

At the end of the second extraction stage, the apparatus again uses the aforementioned solvent reduction mechanism to automatically concentrate the extract to a small volume ready for recovery. Condensed, removed solvent is collected within an internal recovery tank for subsequent purification (recycling) or disposal.

This apparatus was used to carry out extractions according to EPA SW-846 Method No. 3541 with good results. A variety of compounds of environmental interest, such as chlorinated pesticides and other semivolatile analytes, were efficiently extracted from different matrices. Principles of operation were discussed. Recovery and precision data for the target analysis were presented.

### On-line preconcentration, cleanup and HPLC determination of chlorophenoxy acid herbicides in water

*Luz E. Vera-Avila, C. Patricia Padilla, G. Maira Hernandez and L. Jose Luis Meraz, Depto. de Química Analítica, Facultad de Química, Universidad Nacional Autónoma de México, México D.F. 94510*

A selective and sensitive method based on on-line precolumn technologies has been developed for the determination of chlorophenoxy acid herbicides at low μg/l concentrations in water.

The sample containing the herbicides 2,4-D, 2,4-DB, 2,4,5-T, Silvex MCPA and Mecoprop is first filtered through a nylon membrane to eliminate suspended solids. The sample bottle is then rinsed with methanol and pure water pouring all liquids successively through the same filter. Extraction and preconcentration of analytes are carried out by percolation of the mixture through a small 20 × 2 mm I.D. precolumn packed with a polymeric reversed-phase adsorbent. In this step, inorganic interferences are eliminated but many other organic compounds of low and medium polarity remain adsorbed on the reversed-phase packing. Further sample cleanup is performed by a selective on-line transfer of ionized analytes to a second precolumn, packed with an anion exchanger, using an acetonitrile-sodium hydroxide solution. Finally the anion-exchange precolumn is coupled to an analytical reversed-phase C-18 column via switching valves and the chlorophenoxy acids are separated and analysed by gradient elution and UV detection at 230 nm.

The proposed methodology allows the simultaneous determination of the six herbicides at trace levels in drinking water, tap water and surface water. Good recoveries, $\geqslant 90\%$, and high precision, $\leqslant 10\%$, are obtained for all analytes. Sample handling is minimal and analysis time is reduced by comparison to currently used methods based on liquid–liquid extraction, solute derivatization and GC analysis.

### Automation of environmental immunoassays

*C. B. Shumate, Hamilton Company, 4970 Energy Way, Reno, NV 89520*

Enzyme-linked immunosorbent assays (ELISAs) for pesticides and herbicides in environmental and agricultural samples are quickly demonstrating their importance in screening applications. Traditional chromatographic methods are expensive and require extended turnaround times for results, making them incompatible with rapid on-site decision making. ELISA methods have proven to meet or exceed the performance of gas chromatography. Often sampling frequency is limited by the time and cost of these traditional methods. ELISAs offer rapid low cost analysis thereby increasing the frequency of sampling and enhancing data quality. Automated ELISA workstations allow the full benefit of these kits to be realized. Sample preparation, reagent pipetting, incubation, and even photometric evaluation can be performed without user intervention. Reliability is increased through the elimination of operator error, better accuracy and precision, and often higher speed. Much larger batch sizes are possible and these systems can provide sample tracking with report generation for documentation requirements. Statistical performance was presented in light of manual procedures and insight into the critical aspects of automating these ELISA kits was discussed.

### Adaptive nets, cellular automata, and all that stuff: some new, nonlinear methods for extracting information

*Steven D. Brown, Department of Chemistry and Biochemistry, University of Delaware, Newark, DE 19716*

Artificial neural networks are becoming increasingly commonplace in applications in many areas of analytical chemistry. But neural networks are just one manifestation of the range of nonlinear methods now being developed for extraction of information from data. If one considers multilayer perceptron neural networks from a more general perspective than is usually taken, it is possible to see a short distance into the future of nonlinear data analysis.

The basis for—and possible applications of—several extensions of current multilayer perceptron technology were the primary focus of this paper. Several unusual schemes for nonlinear data analysis were introduced. These include adaptive networks, recurrent networks and cellular automata. The use of adaptive multilayer perceptron nets will illustrate the current state of the art of data analysis. Related recurrent networks were discussed and suggested for solution of problems involving time series data, including process monitoring, transient detection, and control. Ensembles of recurrent networks permit much more sophisticated inference and control. A network made from interconnected units which are themselves recurrent neural networks is known as a cellular automaton. Automata may have applications in predicting chaotic time series and in detection of small, transient signals in the presence of larger amounts of noise.

### Genetic algorithms and their applications to analytical chemistry

*Edward V. Thomas, Sandia National Laboratories, Albuquerque, NM 87185-0829*

Genetic Algorithms (GAs) comprise a family of evolutionary search procedures, based on the mechanics of natural selection/genetics, and are used to solve general optimization problems. Unlike traditional methods of optimization, GAs have been shown to work well over a broad range of difficult problems.

In chemistry, GAs have recently been applied to a number of very complex problems. After a brief overview of where GAs have been applied in chemistry, this paper focused on a GA-based procedure for selecting analytical wavelengths when analysing complex materials by spectroscopy. This procedure, which is totally data driven, is simple yet very computing intensive. The use of this procedure was illustrated by a reanalysis of a data set from the literature.

### Automated analysis of polyols

*Frank A. Dethomas and Paul Dallin, NIR Systems, Inc., 12101 Tech Road, Silver Spring, MD 20904*

Polyols are long-chain polymers which can be produced through reactions involving organic oxides, acids and multi-functional alcohols. Uses for the polymeric materials include polyurethanes, surfactants, paint additives, adhesives, and foams. The particular end use of the polyol is dependent on its molecular weight.

Polyols are produced as batch reactions where hydroxyl number (molecular weight), acid value and moisture are determined by titration for the desired endpoints. Near-infra-red spectroscopy has been used for a number of years to monitor the production of polyols. Near-infra-red (NIR) spectroscopy offers a quick, non-destructive method of analysis.

The authors described a new sample presentation device with added automation, simplified operation and improved measurement precision. The presentation focused on new design improvements over current methods and analytical results. Details on spectral regions, sampling methods, optimal pathlengths, temperature correction, and calibration schemes were given.

### Automation of tablet dissolution with an HPLC finish from sample collection to reporting with a validated system

*Allan Macdonald and Donald R. Fourby, Thermo Separation Products, Fremont, CA 94537*

As drug formulations become more complex and regulatory agencies require more active ingredient specific information, the need for using an on-line HPLC finish in tablet dissolution testing has increased. The authors have developed an automated system for tablet dissolution using validated components from the sampling device to the chromatography system and the system software. Control of the analytically sensitive steps in the process was demonstrated in this paper. HPLC system suitability control may be imposed before analysis or collection of the samples. USP, EP, and FDA requirements for sampling times are met even at the shortest sampling times. Sample-to-sample carryover is less than 0.5%. Sample delivery volumes are reproducible to 25 μl.

Both the sample collection and the LC analysis use the same PC-based software to directly link the chromatographic results to the tablet dissolution experiment. The data are then transferred via DDE to the report generation software to complete the direct link to the experiment. The flexibility of the system was illustrated by dissolution of a multicomponent analgesic in both single sampling and multiple sampling modes.

### Automated solid phase extractions with direct HPLC injections for analysis of free cortisol in urine

*Dennis D. Blevins, David O. Hall and Roger Q. Roberts, ANSYS, Inc., 2 Goodyear, Irvine, CA 92718*

Monitoring urine free (unconjugated) cortisol is important as a diagnostic procedure for Cushing's syndrome. The clinical laboratory requires a rapid throughput, and cost effective procedure to perform the analysis. SPEC solid phase extraction (SPE) columns provide clean extracts which can be directly injected into the HPLC without an evaporation step. Interfacing automated devices for SPE allows the laboratory to process specimens unattended.

The presentation reported on work completed, discussing the advantages of the SPEC microcolumn integrated with automated processing equipment. With the reduced bed mass, smaller solvent volumes are required to process the specimen as compared to conventional packed columns. The extraction method allows for direct injection of the elute into the HPLC, and does not use chlorinated solvents or require an evaporation step.

For the automated protocol, pretreated specimens are loaded into test tubes and placed in the sample rack. The SPEC microcolumn discs are conditioned and the sample applied. After the wash step the microcolumns are eluted with 300 μl of HPLC mobile phase into a clean test tube. An aliquot is injected into the HPLC using the injection valve in the Gilson ASPEC processor unit. The method was evaluated using standards, controls, and authentic patient specimens.

### Laboratory robotics—an automated tool for preparing ion chromatography calibration standards

*James L. Chadwick, Westinghouse Electric Corporation, Bettis Site, P.O. Box 79, West Mifflin, Pa. 15122*

This paper described the use of a laboratory robot as an automated tool for preparing multi-level calibration standards for on-line ion chromatography (IC) systems. The robot is designed for preparation of up to six levels of standards, with each level containing up to 11 ionic species in aqueous solution. The robot is required to add the standards' constituents as both liquid and solid additions and to keep a record of exactly what goes into making up every standard.

Utilizing a laboratory robot to prepare calibration standards provides significant benefits to the testing environment. These benefits include:

(1) Accurate and precise calibration standards in individually capped containers with preparation traceability.
(2) Automated and unattended multi-specie preparation for both anion and cation analytical channels.
(3) The ability to free up a test operator from a repetitive routine and re-apply those efforts to test operations.

The robot uses a single channel IC to analyse each prepared standard for content and concentration. These results are later used as a measure of quality control.

System requirements and configuration, robotic operations, manpower requirements, analytical verification, accuracy and precision of prepared solutions, and robotic downtime were discussed in detail.

### Quantitative analysis of remote sensing FTIR interferogram data

*Mutua J. Mattu and Gary W. Small, Center for Intelligent Chemical Instrumentation, Department of Chemistry, Ohio University, Clippinger Laboratories, Athens, OH 45701*

Fourier transform infra-red (FTIR) sensors have been used in various remote sensing environments such as monitoring at hazardous waste sites, leak detection at chemical plants, and regulatory monitoring of smokestack emissions. These sensors also have potential use in clinical applications such as monitoring of glucose levels in diabetic patients and for process monitoring and control in the chemical industry. In each of these applications, a premium is placed on the degree to which the sensor is compact, rugged, and reliable.

To address these requirements, the authors' laboratory is developing data analysis methodology based on the application of narrow bandpass digital filters directly to short FTIR interferograms. With the design of digital filters that pass only the frequencies corresponding to a specific analyte band, spectral information can be isolated directly from a short interferogram. Direct use of the interferogram segment eliminates the need for a separate spectral background measurement, while reducing the length of travel of the moving mirror of the interferometer increases the ruggedness of the spectrometer.

The above scheme has been tested with aqueous glucose solutions which feature significant overlap between the absorption bands of glucose and water. In this case, the filtered interferogram contains signals due to both glucose and water. Multivariate calibration methods were used to overcome this signal overlap. Preliminary results based on the application of partial least-squares regression to 200-point interferograms collected from glucose solutions have yielded three-factor calibration models with $R^2$ values in excess of 99%. In the work presented, the same scheme was used with active bistatic and passive terrestrial FTIR remote sensing data.

Application of digital filtering and pattern recognition techniques to interferogram-based FTIR qualitative analysis has been demonstrated. The feasibility of quantitative analysis of similar remote sensing data was presented. Problems that hinder quantitative analysis of FTIR remote sensing data include the varying temperature of the infra-red source, changing pathlength of the absorbing species, and atmospheric attentuation of the infra-red radiation. These issues were explored using both laboratory FTIR data that simulates conditions found in remote sensing measurements, as well as data collected during actual field trials.

### A new chemometrics environment for FTIR

*Joseph McGuire and Bruce McIntosh, KVB/Analect, 9420 Jeronimo Road, Irvine, CA 92718*

Advanced chemometric processing of infra-red and near-infra-red spectra has dramatically improved the capability of monitoring systems to provide useful process control data. This paper described a new chemometrics calibration environment having characteristics very useful for the easy and accurate development of FTIR and FTNIR calibrations. Application examples were provided to illustrate each of the following topics.

*CPSA algorithms*—The core chemometrics algorithm was developed by Exxon Research and Engineering to provide the following capabilities not found in conventional PLS or PCR calibration packages:

Eliminates baseline drift effects on cal./prediction results
Eliminates path-length change effects on cal./prediction result
Eliminates known interferences such as $H_2O$ vapour on cal./prediction results
Creates 'better' regression coefficients based on step-wise regression
Reduces number of factors used to build model based on above eliminations

*Windows environment*—Windows has become a standard for modern instrument control. The standard Windows Graphical User Interface (GUI) and on-line hypertext help facilitate rapid learning of new programs, and the Windows conventions and tools provide easy sharing of data between programs. For example, the input setup uses standard spreadsheet conventions so data from an experiment plan spreadsheet can be cut and pasted into the calibration without re-entry of experimental data. Likewise, results in tabular or graphical form can be cut and pasted into a word processor to generate a detailed calibration report, again without reentry of results or recreating charts.

*Preservation of spectral formats*—All spectral data including abstract factors, correlation spectra and correction terms are presented with the same X and Y axes defined by the calibration spectra. This allows chemists to apply their valuable visual pattern recognition skills to the interpretation of what the calibration program is actually doing with their spectra.

### Evaluation of a computerized peptide sequence identification system

*Lijuan Hu, Peter de B. Harrington, Elaine Saulinskas and Peter Johnson, Ohio University, Center for Intelligent Chemical Instrumentation, Department of Chemistry, Clippinger Laboratories, Athens, OH 45701-2979*

Peptide sequencing instruments are important tools for bioanalytical chemistry. Knowledge of amino acid sequence permits conclusions to be made about protein structure, relationships among different proteins, and also provides data useful for isolation of the structural gene for a protein.

The Applied Biosystems 477A Protein/Peptide Sequencer is based on Edman chemistry, and the N-terminal amino acids are identified as their phenylthiohydantoin (PTH) derivatives by HPLC. The PTH-amino acids are identified by their relative retention indices. The onboard software uses the succeeding cycle to identify the current amino acid according to the corrected lag. However, misidentification often occurs due to the incomplete cleavage of the N-terminal amino acids. The error will propagate through succeeding peptide cleavages and cause background peaks that must be screened from the identification. Therefore, manual interpretation of the chromatographic runs is frequently required.

An intelligent sequence analysis algorithm has been developed. The algorithm is a fuzzy expert system that uses heuristic rules developed from peptide sequencing experts. The current amino acid is identified by using both the previous cycle and the standard run besides the succeeding cycle. The expert system is applied directly to the chromatographic data from the sequencer. This system was compared to a human expert and the peptide sequencer onboard software.

### Chromatography ChemStation software for the multi-technique laboratory

*Richard C. Gearhart, Gary Jackoway and Thomas J. Stark, Hewlett-Packard Company, Little Falls Site, 2850 Centerville Road, Wilmington, DE 19808*

Hewlett-Packard's Chromatography ChemStation allows complete control of an analysis from sample introduction through study summary reporting. The GC method developer wields totally integrated control of all setpoints for the HP 6890 GC affording unsurpassed capabilities for method documentation and automated optimization. Similar capability for the HP 5890 GC, CE, and HPLC instruments afford the multi-technique lab maximum efficiency in method development and production analysis. The lab manager deploys personnel across techniques with minimal retraining due to the single user interface with common but customizable data analysis and reporting.

Report formatting and output is very flexible and includes several standard formats including GLP and system performance. The user can select system suitability reports as well as sample sequence summaries. For labs requiring additional customization, a report layout designer is standard in the software.

The HP Chromatography ChemStation Software was designed for flexibility but with the requirements of regulated environments fully in mind. User access levels with passwords are available to prevent accidental data loss. System performance can be monitored via run or sequenced based system suitability reporting. On-site user performed software validation capability is a standard feature. Another standard feature, the GLP save register is a binary read-only record of the results of analyses including both the sample data and the method.

The optional HP ChemStore Database allows highly flexible search and reporting capabilities of results including chromatogram graphics and control charts. Case studies of GC and LC data were presented demonstrating the capabilities of the HP Chromatography ChemStation, HP Instrumentation, and HP ChemStore reporting.

### Development of robust separations using a computer-aided method development system

*Jeffrey X. Duggan, Yury Rozenman and Michael Dong, Perkin-Elmer Corporation, 761 Main Avenue, Norwalk, CT 06859-0250*

The process of HPLC method development has been greatly facilitated by modern computerized instrumentation, and a number of approaches are now being used for computer-aided solvent optimization. The Turbo Method Development system described here has been specifically designed for method development. This system consists of a quaternary gradient pump, a variable volume autosampler and a UV diode array detector controlled by the PC-based Turbochrom software and equipped with an integrated software system designed to help solve separation problems.

The software uses both automated four solvent search capabilities and chromatographic simulation to approach method development systematically while the system performs iterative labour-intensive tasks. Turbo Method Development uses a graphic interface to design, execute and interpret specific searches of quaternary solvent space. The results are interpreted through a colour-coded resolution map which is scored based on the number of resolved peaks and the resolution of the critical pair in each separation. Interpolation capabilities allow the exploration of space between actual experiments through chromatographic simulation. The Turbo Simulation software uses input from two or three preliminary runs to predict separation quality over a wider range of solvent conditions.

In this work the Turbo Method Development system was used to develop a stability indicating HPLC method for the separation of the antibiotic lomefloxacin from several of its photodegradation products. The Turbo Simulation software was first used to study the possibility of a binary isocratic mobile phase for this separation. Simulations showed that the problem could be solved isocratically, but that the initial binary solvent system could not resolve all of the components. Turbo Method Development was then used to test various isoeluotropic ternary and quaternary solvent systems. This study revealed that a ternary system containing buffer with ion pairing reagent plus ACN and THF solved this separation problem. The purity and identity of each peak in the final separation was corroborated using the PDA with spectral peak purity and library search capabilities.

In this example a difficult separation was achieved in a minimum amount of time, because all of the time consuming processes were automated and could be run overnight. Resolution maps and chromatographic simulation were used after the runs as interpretive tools both to suggest more focused experiments and to quickly locate the best separation conditions from data containing many runs.

### HPLC automation and data management software based on Microsoft Windows NT

*Rick Gard and Andre Szczesniewski, Hitachi Instruments, Incorporated, 3100 N. First Street, San Jose, CA 95134*

HPLC system capabilities have evolved very rapidly in the last few years. In the recent past, a sufficient system setup would typically include at most an isolated data station designed to setup and control the HPLC hardware; then acquire and process the detector signal. Now these rudimentary operations are assumed, even taken for granted. Desktop computers are now more powerful than ever, and in combination with modern 32-bit multitasking operating systems, such as Microsoft Windows NT, it is now possible to provide a host of extended features that go beyond just the convenience of a standard graphical user interface.

It is now possible to do much more than automatically generate, process, and archive HPLC data. Extended capabilities include such things as providing information about the quality and integrity of the data generated. Furthermore, data need not be isolated inside a standalone workstation or application. Features such as built-in networking and dynamic data exchange with spreadsheets and word processors, allow complete flexibility to produce custom calculation, reporting, and routing of data.

This paper described how this software is employed to provide automated confidence checking of the HPLC hardware components, and maintain password protected file security and audit trails on all data generated. The result is the production of high quality data that is secure, validated, and conforms to GLP.

### Development of automated SFE methods for gravimetric determinations

*Joseph M. Levy, Robert M. Ravey, Victor Danielson, Athos C. Rosselli and Anita Cardamone, Suprex Corporation, 125 William Pitt Way, Pittsburgh, PA 15238*

Supercritical fluid extraction (SFE) has been utilized for the gravimetric determination of fats in manufactured food products (i.e. animal feed and snack foods). Current methodology commonly relies on exhaustive extraction with liquid solvents such as chloroform, hexane, and petroleum ether. Attempts to eliminate the use of toxic, flammable solvents, to reduce conventional extraction times (i.e. 12–16 h), and have an independent monitor of fat levels has prompted food manufacturers to search out practical alternatives. Near infra-red spectroscopy has been used for fat determinations in food, but requires frequent calibrations and updating. SFE presents itself as a practical alternative for fat determinations and can serve to complement near infra-red data. Employing non-toxic, flammable solvents, namely supercritical carbon dioxide ($CO_2$), SFE can be used, due to the physical properties of supercritical fluids, to provide precise, rapid fat extractions without the need for liquid solvent disposal. Moreover, due to recent instrumental developments, especially in decompression control, SFE can be performed reliably and in a very straightforward, routine fashion. This kind of ease of operation is important, for example, for food manufacturing plant laboratories. With supercritical $CO_2$, fats can be extracted in less than one hour in a reproducible fashion, either manually or sequentially automated. This paper described the procedures employed for SFE method development.

### An on-line application of a total organic carbon analyser for the detection of low-level organics in ultrapure water

*Karl D. Brinkman, OI Analytical, P.O. Box 9010, College Station, TX 77842-9010.*

With the increasing demands for lower levels in the detection of organic carbon in the pharmaceutical, semiconductor, and power generation industries, the need for an accurate and fast total organic carbon (TOC) analyser to reach these lower levels is also increasing. A product that conforms to Standard Methods, EPA, and ASTM guidelines is commercially available to reach these lower levels. Using the persulphate at 100°C method for detecting TOC, this analyser is able to perform an analysis quickly, precisely, and on-line. This presentation provided data showing this precision and diagrams detailing the configuration of the analyser and the water system used to produce ultrapure water.

### Continuous TOD monitoring of plant process water for control of chemical and biochemical oxygen demand discharge parameters

*Thomas D. Baugh, E. I. DuPont De Nemours Inc., DuPont Corporate Center for Analytical Sciences, Ponca City Laboratories, 1000 S. Pine, Ponca City, OK 74602-1257 and Thomas A. Jacobi, E. I. DuPont De Nemours, Beaumont Works, Highway 347, Beaumont, TX 77704*

In 1992, to upgrade wastewater facilities at the DuPont Beaumont Texas site and in preparation of anticipated regulations, the plant settling pond was closed as part of a larger plantwide initiative. This pond had previously served to remove suspended solids and biochemical oxygen demand (BOD) from wastewater before discharge. This paper focused on the issue of ensuring compliance with the plants NPDES (National Pollutant Discharge Elimination System) permit in the area of oxygen demand after pond closure.

Fast, on-line analysis of oxygen demand is necessary to detect upset conditions and allow for stream diversion during these times. A laboratory programme assessed the response of likely upset or spill components to the oxygen demand parameters TOD, COD and BOD as well as to total organic carbon (TOC) analysis. Results showed that TOD correlated well with the regulated parameter COD, that BOD could be anticipated if the response of the most sensitive compound were assumed and that TOD was superior to TOC analysis in the case of volatile organic materials which may purge from the on-line TOC before analysis or in the case of hard to oxidize materials.

The installation of four on-line TOD analysers in the period of 1992 through 1994 allows the plant and individual contributors to the plant outfall to control the wastewater system and divert streams as necessary. In the installation and testing phase of the on-line instrumentation, a solids handling system was developed so that TOD could be measured in waters with heavy solids problems. The TOD analysers further have helped to track down persistent subtle leaks of material into the wastewater which would have otherwise gone undetected. Comparison of TOD and COD results led to a change in laboratory COD methodology when unexpected high chloride levels yielded elevated COD values while TOD levels remained stable.

There are also lessons to be learned in interpretation of TOD responses. Since on-line TOD and TOC units analyse filtered samples, any oxygen demand contained in the solids will be missed by these analysers but picked up by laboratory COD measurement. Increases in COD not paralleled by TOD and TOC probably indicate chloride interferences in COD analysis while a TOD increase without increase in TOC indicates the presence of inorganic species in general.

This paper presented an overview of the laboratory data used to validate use of TOD as part of the plant's wastewater control strategy; historical process data in which COD, TOD, and BOD data were compared for the on-line instruments. Examples of how TOD helped plant operations by indicating a persistent subtle leak and by pointing to the need for COD method improvement were presented, along with examples of some of the challenges in interpretation of TOD data.

### A custom automated biochemical oxygen demand system

*Harry M. Harada, Jr., Shian-Shien Kung and Gary D. Peters, Metro Wastewater Reclamation District, Denver, CO 80229*

The Metro Wastewater Reclamation District (Metro) laboratory sets up and reads out around 100 biochemical oxygen demand (BOD) analyses (200 bottles) each day.

The District acquired and is implementing a custom automated BOD system to increase quality and efficiencies, reduce errors, and handle the increasing workload of a wastewater laboratory.

Acquisition and implementation followed project management techniques. Close co-operation among Metro, Biomedical Devices Company (BDC) and Hewlett-Packard (HP) is producing a unique system to handle BOD and carbonaceous BOD (CBOD) workload more efficiently and effectively.

An HP ORCA robot and BDC custom workstations perform the majority of repetitive steps for the BOD analysis. ORCA's Method Development Software coordinates all system operations. The ORCA moves standard BOD bottles for and after processing by the workstations which: remove water seals and bottle stoppers, add seed and/or nitrification inhibitor, add dilution water, acquire dissolved oxygen measurements, replace bottle stoppers and add water seals to stoppered bottles. Simultaneous operations by the workstations allow high throughput for setup and readout phases of the analysis. A load cell ensures accurate dilutions and detects human errors in the setup phase of the procedure. The D.O. metre and probe undergo periodic calibration throughout a run, and are automatically recalibrated as needed.

Experience supports a conviction this design either now does, or will, meet or surpass the goals. Current data indicate slight improvements in precision and accuracy. Calculation and transposition errors and errors in adding seed and inhibitor have essentially been eliminated. Efficiency is noticeably improved. A continuous improvement team is working on further improving the system's performance. The District and BDC are continuing to co-operate in improving the system. Planned enhancements include a faster D.O. probe, remote communications of system error, and an interface to the LIMS.

### Simultaneous determination of total nitrogen and carbon in waste water

*Marco Baccanti, Liliana Krotz and Luidi Ragaglia, Fisons Instruments S.p.A, Strada Rivoltana-20090, Rodano (Milan), Italy and Robert Michael, Fisons Instruments, 55 Cherry Hill Drive, Beverly, MA 01915*

The research of new, fast and automatic methods for water monitoring in industrial wastes is a topic of growing interest. Automatic methods for TOC determination available today are widely applied for this purpose but are not completely satisfactory because of the impossibility to simultaneously perform the determination of the concentration of total nitrogen.

Total nitrogen and carbon combustion analysers for solid samples are available and widely applied for many applications in various research and industrial labs.

The method introduced in this paper permits total nitrogen and carbon determinations in waste water at low ppm level by modifying a commercially available analyser (NA2000, Fisons Instruments).

Samples are automatically injected into the quartz combustion reactor over a layer consisting of a mixture of CuO and a platinum-based catalyst. A complete combustion of the organic compounds dissolved in the sample takes place immediately when the vaporized sample reacts with an oxygen enriched helium carrier gas introduced into the reactor. Combustion gases are then swept on a copper layer, on a water trap and are finally separated by a GC column and detected by a TCD. $N_2$ and $CO_2$ peaks are measured and their concentrations calculated.

Experimental results from the determination of N and C and TOC (after acidification of the sample) in samples of waste water, sea water, drinkable water as well as aqueous standard solutions were shown and discussed.

### A compact, lost-cost automated method for determining total N and total P in water

*Stephen Coverly, Bran + Luebbe, Werkstrasse 4, 22844 Norderstedt, Germany*

The traditional Kjeldahl digestion method for sample preparation for total N and P is time-consuming and uses corrosive chemicals. The more recently developed microwave procedures are faster and less hazardous, but the microwave oven is bulky and costly. On-line microwave digestion requires a high-pressure pump and valves to cope with the high pressures generated during digestion. Flow-through microwave digestors have a much slower throughput rate than continuous-flow analysers which could be coupled to them.

A compact unit (30 × 12 × 20 cm) for use with auto-analysers has been developed, which can digest 30 samples per hour and perform a colour reaction with the digestate. By changing the reagents and manifold configuration the same unit can be used for determining ortho-phosphate, nitrate, nitrite, total N, total organic P or total inorganic + organic P. Previous methods were restricted to either N or P.

Recoveries from various organic and inorganic N and P compounds were shown.

### The development of an improved gold trap for use in trace level mercury determination

*David J. Hassett and Charlene R. Crocker, Energy & Environmental Research Center, University of North Dakota, Box 9018, Grand Forks, ND 58202*

A common method for determination of low levels of mercury in environmental samples is by reduction of mercury ions in solution and the concentration of elemental mercury on gold by sparging, followed by thermal desorption and detection by atomic absorption or atomic fluorescence. Although particular attention has been paid to the overall apparatus, less attention has been paid to the gold trapping. The authors' research focused on the gold trapping, using ground quartz with its low coefficient of expansion as the gold-coated support medium. The use of ground quartz nearly eliminated the problem of pressure drops across the columns during heating found in the media of purchased gold traps.

During the research, three variables were studied:

(1) Absolute mass of gold loading per unit mass of support material.
(2) Diameter of tubing in which the gold-coated quartz is contained.
(3) Length of packing (including absolute mass of packing material).

It was found that application of chromatographic principles to the gold-coated quartz trapping medium resulted in significant improvement of thermal desorption of mercury with sharper and larger desorption peaks and shorter desorption times. The particle-size distribution of the ground quartz was measured with particular attention to the selection of as narrow as possible a range, keeping with chromatographic considerations.

The traps were prepared by reduction of gold chloride deposited on ground quartz particles using hydrogen at elevated temperature. As would be expected, lighter loadings of gold provided improved performance with improved turnaround time and slightly improved peak shape. Various loadings were compared, ranging from 0.125 to 1.0 weight per cent gold. The lightest loading provided superior performance as well as cost savings. Although the lighter loading provided superior performance, durability has not yet been assessed. It is anticipated that a heavier coating for the first trap in a double amalgamation setup with a light loading for the analytical trap would be the preferred arrangement.

### Analysis of environmental mercury using on-line microwave digestion with a dedicated flow injection system

*S. McIntosh, C. Hanna and J. Baasner, Perkin-Elmer Corporation, 761 Main Avenue, Norwalk, CT 06859-0219*

Public awareness regarding the toxicity and environmental impact associated with mercury contamination has focused a great deal of attention on the determination of mercury in environmental samples. The detection limits of mercury in these samples has been dramatically improved with the development of a dedicated mercury system utilizing the Flow Injection technique.

The Flow Injection technique automates the generation of elemental mercury provided increased sample throughput with the additional benefits of reduced sample and reagent consumption as well as reducing laboratory waste. When the flow injection technique is coupled with the Perkin-Elmer dedicated Flow Injection Mercury System (FIMS) an instrument detection limit of 4 ng/l Hg is obtained. This level of mercury detection is essential when determining 'chronic mercury levels' in aquatic ecosystems and also provides enhanced detection of mercury in drinking water and sediment samples. Data obtained from the determination of Hg in aqueous and sediment samples using the FIMS were presented.

In the quest for improved detection limits sample preparation for the determination of mercury in environmental samples has frequently been overlooked. Current digestion procedures are labour intensive and lengthy, often requiring a two hour digestion time. The development of an on-line microwave digestion system has resulted in reduction of sample preparation times from hours to seconds, thereby reducing the total time required to prepare and analyse the sample to a matter of minutes. The benefits of using on-line microwave digestion coupled with the flow injection analysis for various environmental sample types were discussed.

### Automation of laboratory data entry systems 'a new model for the small to mid-sized lab'

*Lloyd E. Jacobs, En Chem, Inc., 1795 Industrial Dr., Green Bay, WI 54302*

This paper discussed the approach taken by an environmental laboratory to maximize productivity by automating the data entry process through the use of an integrated software system, run on networked computers.

The downturn in the cost of computers and software has made possible a centralized data system that was once only available on expensive mainframe systems. Instrument manufacturers have standardized on the DOS based computer, making interconnection of systems very easy. Automation of the data entry and reporting process removes a significant burden from the analyst, allowing more time for technical review of data and virtually eliminates transcription and other clerical errors.

The system has evolved into a fully automated combination of hardware and software. The instrumentation is controlled by Chemstation software by Hewlett-Packard (Palo Alto, CA). The data files are stored on a Novell Netware 3.11 file server (Novell, Inc., Provo, Utah), and processed by Conifer LIMS by Telecation, Inc. (Morrison, CO).

### Optimizing LIMS automation and human interaction

*Louise L. McGinley, William Tumbleson, Automated Compliance Systems, Inc., 8834 N. Capital of Texas Highway, Austin, TX 78759 and James N. Bower, Automated Compliance Systems, 245 Highway 22 West, Bridgewater, NJ 08807*

Automation of data processing tasks is an important aspect of implementation of any LIMS. This automation typically begins with the upload of analytical results from laboratory instrumentation, and may include other data processing functions such as calculations, data review and approval, release and reporting of the data, and messaging about work status and results. With a set of appropriate tools, the automation of data processing tasks is accomplished in a modular, procedural, and documentable fashion, providing the user with control over all configuration steps.

Many systems address data automation with a vendor-supplied turn-key approach which provides minimal flexibility for modifications by the end-user. In this kind of system, the user selects a pre-configured instrument driver for their application, and usually relies on vendor assistance with new instrumentation. However, providing the user with powerful automation tools encourages a more hands-on implementation, and less reliance on a single vendor. This allows the user to accomplish more comprehensive automation of data processing functions and extends the automation into more complex aspects of system operation.

Segmenting data automation into discrete layers requires that the LIMS incorporate a comprehensive set of tools to address all tasks in the data processing operation. This technique allows the user to configure and link appropriate data automation steps into a smoothly integrated system. By linking automated and manual functions into an automated chain of events, the LIMS provides maximum utilization of all computer resources. In addition, by maintaining manual processing steps within this chain, the overall traceability of data is protected.

Automation may also be required in areas outside the processing of analytical results. Through configurable instruction sets, the LIMS can trigger actions upon the occurrence of a specific event. By providing a tool for user configuration of instruction sets, the LIMS opens a new arena of automation potential.

### An intelligent portable barcode terminal for a PC-based LIMS

*M. P. Kelly and G. F. Gostecnik, Analytical Automation Specialists, Inc., 11723 Sunbelt Court, Baton Rouge, LA 70809*

Conventional LIMS sample login and data entry workstations (computers) have had the requirement that they be physically attached to the Local Area Network (LAN). This paper reported on the development of a Portable Data Entry Terminal (PDET) which, after being downloaded with the appropriate program, may be disconnected from the workstation and carried to a remote work site. This remote site may be a work area within the laboratory which does not have a workstation or any site(s) outside the laboratory.

At the work site, the analyst is prompted for entries including the site location, any other pertinent sample information and any results that are obtained from tests conducted at the site. Each entry is automatically date and time stamped thus assuring an accurate record of the date and time of sample collection and/or results entry. Information may be entered via a keypad or by barcode. When the PDET is returned to a workstation on the LAN, it is placed in a docking station and all of the information is uploaded automatically to the LIMS.

The PDET may be used within the laboratory to enter results from instruments which are not directly interfaced to the LIMS. This not only serves to avoid the cost of interfacing older instruments which are not amenable to computer interfaces or instruments which are used infrequently, but reduces the opportunity for transcription errors by avoiding long sessions at the keyboard of a workstation entering data from a worksheet.

### A bridge to the future: the integration of older instruments into a LAN-based laboratory

*Brian Anthony, Ken McGreevy and Neerja Raman, Hewlett-Packard, Analytical Products Group, Palo Alto, CA 94304*

Laboratories are increasingly faced with the problem of what to do with older instruments. These instruments still separate mixtures using the concepts of chromatography, but lack the power and ease of use of the technology in modern instruments. The economics of the current marketplace make replacing these instruments cost prohibitive. However, the suppliers of analytical services must provide information faster and more efficiently via newer electronic technologies, such as faxes and e-mail. One of the ways for analytical laboratories to provide this type of service and still retain their existing investment in older instrumentation is to integrate equipment via a laboratory local area network.

LAN-based laboratories are not a new idea, having been around for many years. The difficulty in setting up a LAN-based laboratory is usually the expense of the equipment and the training of the laboratory personnel. When older instruments are tied into the LAN, the laboratory wins in two ways. First, the training curve on older instruments is non-existent. Second, the laboratory gains access to newer technology that the older instruments were never designed for. The ability to use modern data collection systems with access to multimedia and almost instantaneous communications give the laboratory the advantage in the marketplace today.

This paper explored the abilities that exist to plug new server technologies into older investments. The ability to integrate older integrators, balances, and printers into the LAN make the investment in these resources more profitable to the laboratories. These servers are not just another place to store data but a resource that allows the laboratory to make use of communication technologies of today. Examples shown included the transmission of reports automatically via an integrated fax server, and via an easy-to-use interface that connects the laboratory to the information superhighway.

### The artificial-intelligence based LIMS system

*Patrick E. Dessert, Christian C. Wagner, SA Consulting Company, 39526 Westminster, Novi, MI 48375 and Carol J. Kelly and Donald A. Lee, Ford Motor Company, Ford Central Laboratories, 15000 Century Dr, Dearborn, MI 48120*

The Laboratory Information Management System (LIMS) presents a fertile environment for the application of artificial intelligence-based applications. Artificial intelligence (AI) is the field of computer science aimed at endowing the computer with human like capabilities. Reasoning, explanation, learning, and natural language understanding are but a few of the capabilities that AI can provide a computer. In a LIMS system these capabilities can reduce error, preserve corporate knowledge, ease the manner user communicate with the system, improve quality, and optimize throughput time. In this presentation the application of AI in the LIMS system being developed at Ford Central Laboratory was described as follows:

*Intelligent instrument integration*—Analytical instrumentation on the system will perform precision and accuracy studies to validate data at delivery time. The instrumentation will also report its own maintenance needs and requests for preventative maintenance.

*Report generation*—The LIMS system will intelligently configure the final test reports based on the input from the laboratory customers and laboratory findings.

*Self learning routing tools*—The LIMS system will route an incoming lab request to the appropriate person in the lab based on the attributes of the request and the capabilities of the lab resources. The tool will be endowed with a learning capability so it will continually improve and reevaluate its performance over time.

*Test planning*—The LIMS system will help users to generate test plans and the assign tasks. The system will help determine the best test ordering and resource allocation to minimize the overall elapsed time required to satisfy the lab request.

*Monitor and track*—The LIMS system assures that the test plan is being followed reporting deviations to users and management. For example, if a lab request is not assigned to a lab employee within 24 hours receipt, management will be notified to resolve the problem.

These are a few of the AI applications that will be available on LIMS. The benefits of these tools include a significant reduction in the time between sample submission and reporting. The AI based tools provide assurance that reported data meet quality standards for precision, accuracy, and traceability. Thus, the quality of the output is improved, providing the customer with a better product. These benefits act in turn as a revenue generator for the laboratory as the service being provided to customers is of greater value.

### Sample conditioning of wastewater stream for on-line analysis

*Nish Vasavada, Dan Dodson and Randy Lamirand, Eastman Chemical Company, Longview, TX 75607*

Texas Eastman Division (Longview, Texas) of Eastman Chemical Company installed on-line analysers at five wastewater streams in 1993. Monitoring stations are located at key locations in the process sewer network. Each station is equipped with a pH analyser, temperature sensor, automatic sampler and TOC analyser. Ammonia analysers are installed at two of the five locations. Data collected by the analysers is communicated to the distributed control system (DCS) located at the wastewater plant. Applied Automation Hartmann and Braun procured and installed the analysers as per Eastman specifications. The monitoring system has proved to be an excellent tool for each detection of excess carbon load in the process sewer. Part of the wastewater is diverted to stormwater tanks if necessary. This ensures smooth wastewater plant operation and improved treated water quality.

Composition of the wastewater streams at each monitoring station is unique. Presence of oils, chemicals, grit and other impurities pose a challenge for sample conditioning design. Field testing of three different brands of TOC analyser had shown that the success or failure of this project depended on a good sample conditioning system. The sample treatment consists mainly of a 20 mesh (1000 micron) strainer, followed by Collins Model 9000 filtering system. Magnetic coupled agitators provide turbulence for particle removal from the filter surface made of Teflon. The original Teflon filters were replaced later on with wire mesh filtering elements to remove particles larger than 20 microns. Another important change was to supply sample to the Collins filter to process the sample 'on demand' only, which extended the filter element life to four weeks. Sample dilution system is provided to improve TOC analyser's measurement range. A dedicated instrument technician crew does routine maintenance work on the analysers. All stations had been in service for about one year as of December 1994 and were operating very well.

### Remote discrete sampling for process analytical chemistry

*Jimmy G. Converse, Sterling Chemicals, Inc., P.O. Box 1311-R2, Texas City, TX 77592*

The primary reason for applying remote discrete sampling techniques is to provide a means of reaching into a process and extracting a small amount of material for analysis. Continuous sampling systems require that gross quantities of material be extracted from the process which leads to waste and excessive emissions, residue, energy usage, and system maintenance! Why condition a barrel of material when only a drop is required for analysis? We need to extract only the minimum amount of material for analysis to reduce the disposal requirements for cleanup. Any more than the minimum is wasteful.

The main problem is that we have not addressed the *sample preparation* aspect of on-site automated chemical analysis adequately. Traditionally, we tapped the process stream and let it flow through the analyser. As the applications progressed to dirtier and more complex materials, we added gadgets to the sample line to clean up the material. What evolved was miniature chemical processing units that became *expensive to implement and difficult to maintain. They also became the main cause of analyser system failure.* The first rule of sample preparation is that the composition of the material extracted must be representative of the process stream population. *This does not mean that it has to be identical!*

This presentation discussed the fundamental principles of remote discrete sampling utilizing injection into a flowing carrier and multidimensional sample preparation techniques. Application of several preparation operators, such as column separation, vapour liquid equilibrium, chemical reaction, membrane barriers were presented. Examples of handling lethal, corrosive, and otherwise difficult materials were given. Injection of an internal standard allows the entire system performance to be monitored and SQC charts to be generated on a real time basis for analyser system validation.

### Sampling systems for process mass spectrometry: overcoming barriers in placing production instruments

*Larry G. Wright, Mark A. Lapack, Wayne W. Blaser, The Dow Chemical Company, Analytical Sciences, Building 1602, Midland, MI 48667 and Chris Lynch, The Perkin-Elmer Corporation, Real-Time Systems Division, 50 Danbury Road, MS/201, Wilton, CT 06897-0300*

Tremendous pressure exists in chemical manufacturing to maximize capacity and yields, while reducing production costs in order to be a low-cost supplier. In order to meet these increasingly stringent demands, timely and reliable analytical information is needed during production to control the manufacturing process and ensure that the process adheres to strict environmental laws. Many of these analytical demands are being met through the use of on-line mass spectrometers, especially for those applications where continuous monitoring with high sample throughput, speed, dynamic range, and sensitivity is a prerequisite. Continuous on-line analysis has benefits over conventional 'laboratory grab sample methods' in that it provides the required analytical results about specific constituents of a sampled process in real-time without compromising data quality. Based on immediate and continuous process data, a production plant is able to: (1) operate the process under conditions at which optimum yields can be achieved; (2) assure that optimum conditions are met during manufacturing; and (3) quickly respond to conditions that exist when process upsets occur, yields reach unacceptable levels, or conditions in the manufacturing area threaten the safety of workers or the environment. Another benefit of using this type of analyser is that it spreads investment costs by single-handedly providing multiple component and/or multiple stream analyses—a capability that is unavailable to many analytical technologies due to sample selectivity and throughput requirements.

While much of the success of an on-line instrument can be attributed to the accuracy and precision of the analyser, the true success of an on-line system often hinges upon the integrity of the sample interface which directly links the analyser to the process stream. This interface must be dependable and preserve the integrity of the sample. In fact, it is the combination of the analyser with the sample system working in tandem that makes an on-line instrument a reliable tool for consistently providing manufacturing with high quality data. Innovations in sampling such as membrane samplers, dynamic headspace samplers, proportional pressure controllers, multipoint sampling valves, etc., that are making mass spectrometry more amenable to monitoring different types of production streams were described.

### Gas monitoring with fibre optic evanescent field sensors

*B. Mizaikoff, R. Götz and R. Kellner, Institute for Analytical Chemistry, University of Technology Vienna, Getreidemarkt 9/151, A-1060 Vienna, Austria*

Due to the increasing demand for ways of monitoring chemical species continuously, the field of chemical sensors has become important. The introduction of a new generation of IR transparent materials, such as silver halide (AgBr/AgCl) and sapphire ($Al_2O_3$), gives access to a broad spectral region from 3500 to 500 $cm^{-1}$. Associating these new fibre materials with fibre optic evanescent field absorption spectroscopy (FEFA) has led to the development of fibre optical chemical sensors with the ability of analysing many analytes in the aqueous phase and in the gas phase.

In this paper the authors discussed recent advances in the field of gas analysis and gas monitoring. The investigation of a variety of gaseous halogenated hydrocarbons with a sensor system based on polymer coated silver halide fibres was presented. Furthermore, the results were compared with gas chromatographic measurements. In order to optimize the polymer layer on the fibre surface, the diffusion behaviour of the halogenated hydrocarbons into different polymers was studied.

The new application of sapphire fibres in the field of fibre optic gas sensing systems was also discussed. The chemical, mechanical and temperature stability of these fibres allows fibre optic sensors to be used under rough conditions, such as high temperature.

In both applications, the fibre was coupled externally to a conventional Bruker Fourier transform infra-red (FTIR) spectrometer. The active sensor head was located in a gas flow cell connected with a gas sampling device.

Several gaseous species can be detected at the same time. So, fibre optical chemical sensors based on the FEFA principle provide an accurate and versatile analysis tool in the field of multicomponent gas analysis and continuous monitoring systems.

### Remote monitoring of moisture in nylon production

*Lou Sekula, DuPont-Victoria, Old Bloomington Road, Victoria, TX 77902, and Stephen L. Monfre, NIRSystems, Inc., 12101 Tech Road, Silver Spring, MD 20904*

During the early stages of nylon production, it is important to monitor the moisture concentration to preserve the integrity of the catalyst used during the adiponitrile reaction stage. Ideally this monitoring should occur in the process environment. However, due to the reactivity of the compounds used in the production, measurement locations, and the process environment itself, makes process monitoring of moisture during the early stages of nylon production difficult.

Near-infra-red (NIR) spectrophotometry has been used successfully to monitor moisture during the early stages of nylon production. Unfortunately, most of instrumentation used for the analyses required that the actual analysis be performed through a site glass sample port. Furthermore, these instruments are not multiplexed systems, therefore, one instrument is required for each measurement location, thereby increasing the cost per measurement location.

New developments in NIR instrumentation and fibre-optics has helped to improve the process measurement capabilities of NIR analysis. This presentation focused on those developments, which include multiplexing of signals to reduce cost per measurement point, remote locating of the instrument to centre the sensor between three sampling locations, configuration changes needed to meet environment classification requirements, and the effect of fibre-optic distance (120 feet) on the actual measurement.

### An approach to process optimization in the pharmaceutical industry using a system based on the HACCP concept

*J. M. Singer, 244-09 57th Drive, Douglaston, NY 11362*

The FDA has recently published proposed regulations in the Federal Register of a preventive control system for the seafood industry in accordance with Hazard Analysis of Critical Control Point (HACCP) principles. HACCP is a systematized preventive program of hazard control to ensure food safety. This is based, in part, on the identification of the likely hazards and the points at which they could occur in food processing operations and the implementation of programs to monitor these critical points. Systems of controls based on the HACCP concept are designed to detect and document the occurrence of problems, so that they can be corrected expeditiously.

A comprehensive approach to process optimization has been developed for a pharmaceutical company using the HACCP principles as a framework. The project plan is staged in phases. The first phase starts with an analysis and definition of the present state. This includes a series of detailed flow charts tracing the entire operation from the procurement of ingredients to the packaging of the finished dosage form. Analytical test methods are detailed and compared against product registrations; manufacturing procedures are similarly treated. The second phase involves statistical data analysis, identification of issues and opportunities for improvements or enhancements. The last phase entails validation, updates to product registration and/or compendia and subsequent implementation. Once the entire sequence is completed it serves as the mechanism for continuous improvement.

This presentation discussed HACCP and focused on its application to a pharmaceutical manufacturing operation. The various principles were described with examples and considerations which need to be taken into account in the planning and implementation of such a system.

### Maintenance-free, multicomponent analyser for monitoring contaminants in wastewater

*Kenneth E. Creasy, Italo A. Capuano and William T. Lefebvre, Olin Corporation Research Center, 350 Knotter Drive, Cheshire, CT 06410*

Economic and environmental realities in the chemical industry are forcing performance expectations of on-line analysers far beyond those of only a few years ago. A reliable process analyser was developed to continually monitor aqueous streams for organic compounds such as benzene, pyridine, and trichloroethylene.

Unlike conventional process chromatographs, this analyser requires very little maintenance. Chemical species from water streams are analysed either directly or by stripping techniques using chromatographic separation and a photoionization detector (PID). Utilization of a PID permits analyser operation with minimal service and maintenance. This fully automated chromatographic instrument runs entirely on existing plant utilities. Electricity, air, and nitrogen are all that is required for this instrument to operate maintenance-free for months. This instrument not only prepares and analyses a sample from an effluent stream, but also cleans itself periodically. In addition, diagnostic sensors located within the instrument ensure that the instrument is functioning properly, and validate each analysis. The analyser is linked to a computer, which provides control, display, diagnostic, and communication capabilities to the instrument. This system has reproducibly detected organic contaminants at levels far below one part-per-billion.

### A study of the on-line analysis of sulphur in reformulated gasoline

*Richard L. Terry, ARCO Products Company, 1801 E. Sepulveda Blvd., Carson, CA 90745*

A three-month study on the on-line analysis of sulphur in reformulated gasoline has been conducted. A commercial Antek analyser was incorporated into an analyser system that is expected to ultimately be used for on-line certification of three grades of gasoline meeting CARB 1995 reformulated gasoline specifications. The data will be used to control blender recipes during the mixing process.

Additions to the system were presented. Precision data gathered during validation studies and normal operations were presented for both on-line and laboratory operations. Quality control materials were analysed on a systematic basis to acquire this data.

Accuracy of both methods was assessed by frequent analyses of standard materials. Control charts showing statistical comparisons between on-line and laboratory techniques were presented.

### Injection systems for process gas chromatography at trace levels

*Somenath Mitra and Yong Hua Xu, Department of Chemical Engineering, Chemistry and Environmental Science, New Jersey Institute of Technology, Newark, NJ 07102*

Gas chromatography has been used in process stream monitoring for the last 40 years. In an ideal continuous monitoring device, the sample should flow continuously through it. In chromatography this is not possible because the sample has to be introduced as an impulse. Consequently process GCs require an injection device that can automatically inject the sample from a flowing stream. Gas sample valves have been widely used as GC injectors, where the typical injection volume can vary between a few microlitres to a ml (for capillary columns). A large injection volume causes excessive band broadening, and a small injection volume reduces the sensitivity.

The authors demonstrated the use of an on-line microtrap (OLMT) as an injection system for process stream monitoring at trace levels. The sample continuously flows through the OLMT which is placed directly ahead of the GC column. The OLMT is packed with an adsorbent which traps the analytes. The analytes are rapidly desorbed by electrical heating of the microtrap. The desorption serves an injection for GC separation. Thus the OLMT serves the dual purpose of sample concentration and injection. As a result, monitoring of very low concentration levels is possible.

**Computer aided column selection and optimization for on-line gas chromatography**

*Richard Villalobos, OnLine Analytics, Box 1742, Duxbury, MA 02332-1742*

Recent enhancements to OnLine GC Lab provide an integrated software package for methods development for gas chromatography applications. A built-in retention index data base permits selection of the optimum phase for the separation. Data is stored in thermodynamic based format, and will accept the user's own lab data or from the literature. Data compilations are available on diskette for loading and augmenting the data base.

For separations that cannot be done on a single phase, the system provides for selection of two phases with dissimilar retention properties for constructing series coupled column systems. Integral window diagrams visualize the optimum ratio of the two phases for maximum separation factors and fastest analysis. User input values for FID sensitivity and detector electronics time constant customize the optimization parameters to the performance of the user's own chromatograph.

The model is based on the Golay–Giddings equation, and model permits to manipulate all column variables, including length, diameter and film thickness, as well as types of carrier gas and operating temperatures and pressure. Calculated retention times and peak widths were reported in table form and visualized graphically in chromatogram form. Typical applications were presented.

